# Detection of brain-directed autoantibodies in the serum of non-small cell lung cancer patients

**DOI:** 10.1371/journal.pone.0181409

**Published:** 2017-07-26

**Authors:** Manoj Banjara, Chaitali Ghosh, Aaron Dadas, Peter Mazzone, Damir Janigro

**Affiliations:** 1 Cerebrovascular Research, Cleveland Clinic Lerner Research Institute, Cleveland Clinic, Cleveland, OH, United States of America; 2 Department of Biomedical Engineering, Cleveland Clinic Lerner Research Institute, Cleveland Clinic, Cleveland, OH, United States of America; 3 Department of Molecular Medicine, Cleveland Clinic Lerner Research Institute, Cleveland Clinic, Cleveland, OH, United States of America; 4 Department of Biomedical Engineering, Ohio State University, Columbus, OH, United States of America; 5 Respiratory Centre, Cleveland Clinic, Cleveland, OH, United States of America; 6 Flocel Inc., Cleveland, OH, United States of America; 7 Department of Physiology, Case Western Reserve University, Cleveland, OH, United States of America; University of Portsmouth, UNITED KINGDOM

## Abstract

Antibodies against brain proteins were identified in the plasma of cancer patients and are defined to cause paraneoplastic neurological syndromes. The profiles of brain-directed antibodies in non-small cell lung cancer (NSCLC) are largely unknown. Here, for the first time, we compared autoantibodies against brain proteins in NSCLC (n = 18) against those present in age-matched non-cancer control subjects (n = 18) with a similar life-style, habit, and medical history. Self-recognizing immunoglobulin (IgG) are primarily directed against cells in the cortex (P = 0.008), hippocampus (P = 0.003–0.05), and cerebellum (P = 0.02). More specifically, IgG targets were prominent in the pyramidal, Purkinje, and granule cell layers. Furthermore, autoimmune IgG signals were localized to neurons (81%), astrocytes (48%), and endothelial (29%) cells. While cancer sera yielded overall higher intensity signals, autoantigens of 100, 65, 45, 37, and 30 kDa molecular weights were the most represented. Additionally, a group of 100 kDa proteins seem more prevalent in female adenocarcinoma patients (4/5, 80%). In conclusion, our results revealed autoantigen specificity in NSCLC, which implicitly depends on patient’s demographics and disease history. Patients at risk for lung cancer but with no active disease revealed that the immune profile in NSCLC is disease-dependent.

## Introduction

Lung cancer is the most commonly fatal type of cancer in both male and female populations [1]. According to “Cancer Facts & Figures, 2016”, it is the second most frequently diagnosed cancer every year (14% male and 13% female) and the leading cause of cancer-related death equally affecting both genders [2].

A wide array of tumor-associated autoantibodies has been identified in lung cancer patients [3]. The breakdown of B cell tolerance towards corresponding autoantigens for the production of autoantibodies is a hallmark of autoimmune disease, which is also typical in cancer [4]. Even though spontaneous humoral immune responses in cancer patients recognize antigens whose expressions are restricted to tumor cells, most cancer-associated antibodies are directed against self-antigens [5]. Interestingly, the repertoire of autoantibodies found in cancer patients partly overlaps with autoantibodies found in patients with autoimmune diseases [5]. Anti-nuclear antibodies associated with systemic autoimmune diseases such as systemic lupus erythematous (SLE), systemic sclerosis (SS), and Sjogren’s syndrome are also detected in cancer patients [6]. More specifically, about 30% of patients with cancer were estimated to have these autoreactive antibodies [7]. The features of SS and SLE patients have been found in cancer patients of various types such as head and neck, breast, colon, gastric, and lung [5, 8].

The immune system is stimulated independently by self or foreign molecules via the activation of specialized antigen-presenting cells, which in turn expresses costimulatory molecules and promotes T and B cell activation [9]. In a large tumor, a proportion of cancer cells is generally exposed to hypoxic and metabolic stress, and is prone to necrotic and apoptotic cell death that can favor the induction of autoreactive immune responses [10]. Since some autoimmune disease-associated antibodies have been proven to induce tissue damage, it is essential to identify autoantibody accumulation sites in the target organs. Paraneoplastic syndromes (PNS) associated with cancer are known to impair various organ functions, including endocrine rheumatologic, hematologic, dermatologic, and neurologic [11].

The presence of several autoantibodies against various elements of the nervous system has been reported in cancer. In paraneoplastic neurological syndromes (PNNS) antibodies are directed against targets such as amphisphysin, Hu¸ Ri, Yo, Tr, collapsing response-mediator protein (CRMP5/POP66), voltage-gated calcium channels (VGCC), nicotinic acetylcholine receptors (AChR), Purkinje cell cytoplasmic antibody (PCA2), n-methyl-D-aspartate receptor (NMDAR), and leucine-rich glioma-inactivation protein 1 (LGI-1) [11–14]. It is important to note that many of these autoantibodies are not unique to individuals with cancer, nor are they found in all cancer subjects. Furthermore, such self-reactive antibodies are often identified in populations with other cancer types including ovarian, breast, renal, and bladder [12].

Although PNNS are linked to many types of malignancies, they are more frequently associated with lung cancer [11]. Most PNNS are seen in small cell lung cancer (SCLC) patients [11], although based on case reports PNNS have been also been reported for patients with NSCLC [15–18]. The immunological profiles of brain-directed antibodies in NSCLC are largely unknown. Since NSCLC, namely adenocarcinoma and squamous cell carcinoma, occur in approximately 85–90% of lung cancer patients, the impact of autoantibodies might have been much larger in this group [19]. Brain-directed autoantibodies and symptoms of PNNS can be detected before cancer symptoms and histological diagnoses. Thus, prompt identification of specific peripheral autoantibody markers and their targets in the CNS has potential applications to cancer diagnosis, early detection, and therapy.

In the present study, we have evaluated the serum of NSCLC patients for antibodies that are directed against brain proteins using immunohistochemical and immunoblot analyses. All previous studies were carried out to detect the presence of antibodies against specific antigens comparing immunoreactivities between patients’ sera and antibodies in control subjects. We instead compared the prevalence of brain-specific antibodies in cancer patients with age-matched non-lung cancer controls with similar medical history, risk factors, and demographics.

## Materials and methods

### Patients

The study protocol followed the guidelines of the Declaration of Helsinki, and was approved by the Institutional Review Boards of the Cleveland Clinic Foundation. All subjects enrolled in the study provided written informed consent according to the institutional review protocols. Patients' demographic information is summarized in Table 1. Subjects were separated into two groups: 1) non-small cell lung cancer (NSCLC), as confirmed by histology (cancer, can; N = 18) and 2) similar age, habit, and lifestyle, but not-cancer (control, con; N = 18). The serum samples obtained from cancer patients, age ranging from 49 to 88 years (median = 70.6 years, 66.6% males, 55.6% lung adenocarcinoma) were compared with control patients, age ranging from 51 to 76 years (median = 62.8 years, 55.6% males) for immunoglobulin reactivity to rat brain antigens.

**Table 1:** Patients’ demographic properties.

Patient ID	Age	Gender	Smoking History	COPD	DM	Histology	Stage
Con 1	59	F	Former	N	N	N/A	N/A
Con 2	68	M	Former	N	N	N/A	N/A
Con 3	70	M	Current	N	N	N/A	N/A
Con 4	65	M	Former	Y	N	N/A	N/A
Con 5	76	M	Former	Y	N	N/A	N/A
Con 6	55	F	Current	N	N	N/A	N/A
Con 7	54	F	Former	N	N	N/A	N/A
Con 8	51	M	Current	N	N	N/A	N/A
Con 9	58	F	Former	N	N	N/A	N/A
Con 10	60	F	Current	N	Y	N/A	N/A
Con 11	52	M	Former	N	N	N/A	N/A
Con 12	68	M	Current	N	N	N/A	N/A
Con 13	58	F	Former	N	N	N/A	N/A
Con 14	69	M	Former	N	N	N/A	N/A
Con 15	68	M	Former	N	N	N/A	N/A
Con 16	73	F	Current	N	N	N/A	N/A
Con 17	58	F	Former	N	N	N/A	N/A
Con 18	69	M	Former	N	N	N/A	N/A
Can 1	58	F	Former	Y	N	Adeno	Ia
Can 2	72	M	Current	Y	N	Squam	IIIa
Can 3	63	M	Former	N	N	Adeno	Ia
Can 4	70	M	Former	Y	N	Squam	IIb
Can 5	74	F	Former	N	N	Adeno	Ib
Can 6	54	F	Former	N	N	Adeno	Ib
Can 7	86	M	Former	Y	N	Adeno	Ia
Can 8	63	F	Former	Y	Y	Squam	IIb
Can 9	49	M	Former	N	N	Adeno	IIb
Can 10	88	M	Former	N	N	Squam	Ib
Can 11	79	M	Former	N	N	Adeno	Ia
Can 12	77	M	Former	N	N	Squam	IIb
Can 13	78	M	Former	Y	N	Adeno	Ia
Can 14	83	F	Former	N	N	Adeno	IV
Can 15	80	M	Former	N	Y	Squam	IV
Can 16	65	M	Former	Y	Y	Squam	Ib
Can 17	76	F	Former	N	N	Adeno	IIIa
Can 18	57	M	Former	N	N	Squam	IIIa

Con: Control, Can: Cancer, M: Male, F: Female, COPD: Chronic obstructive pulmonary disease, DM: Diabetes mellitus, Y: Yes, N: No, N/A: Not applicable, Adeno: Adenocarcinoma, Squam: Squamous cell carcinoma

### Serum samples

**Fig 1:**
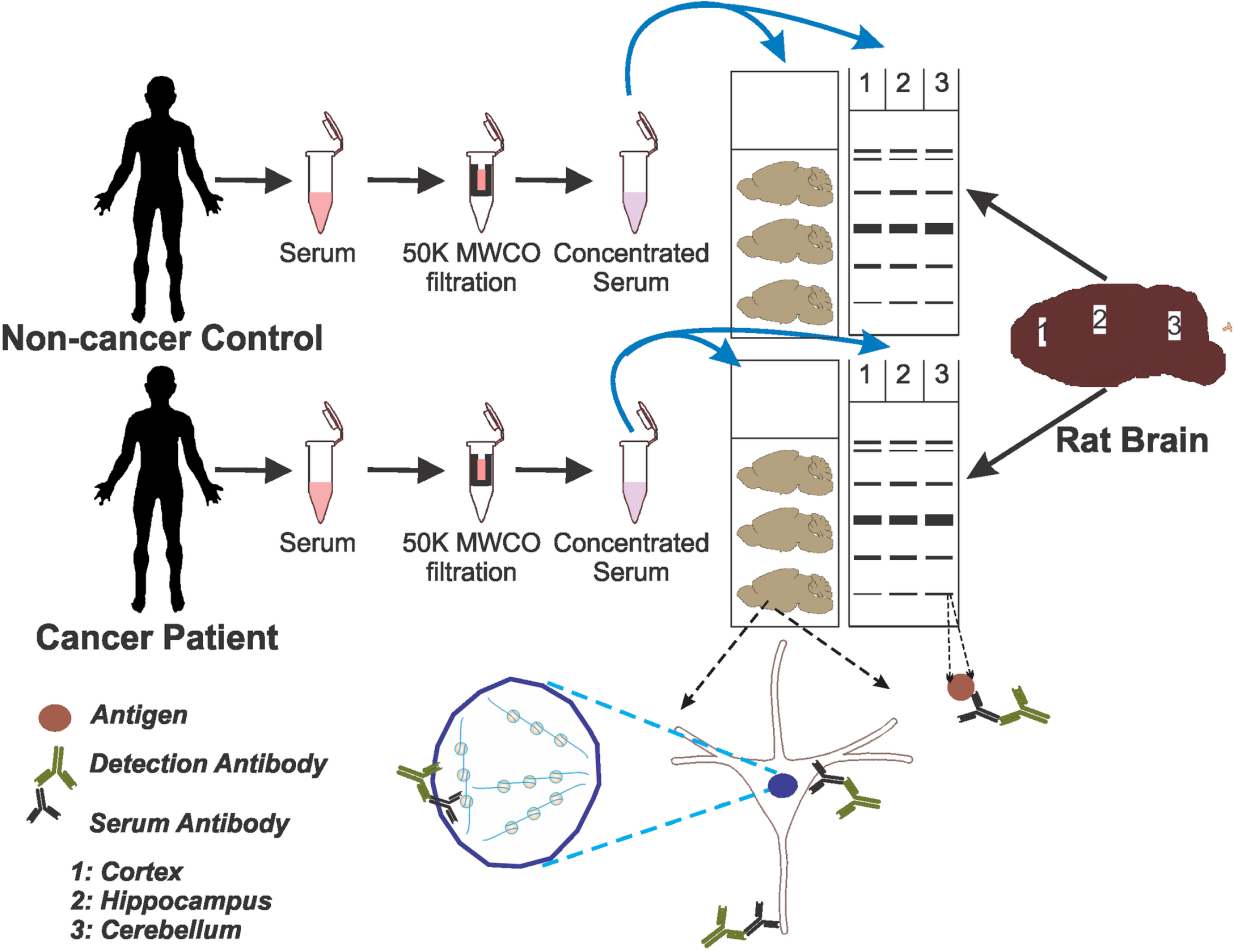
Schematic representation of the experimental design. Sera from lung cancer patients and non-cancer control individuals with similar lifestyles, habits, and age were filtered in 50 kDa molecular weight cut-off (MWCO) centrifugal filter devices to reduce small molecular weight proteins. These sera were then evaluated by immunohistochemistry and Western blotting performed on rat brain tissue and protein extract, respectively. Rats were sacrificed after intracardiac perfusion with PBS. Brains were then sectioned or dissected to harvest cortex, hippocampus, and cerebellum. Tissues were used for immunohistochemical detection to produce the results shown in Figs 2–4, whereas total protein isolated from the brain regions were analyzed by Western blotting as shown in Fig 5. The specific reactivity measured in Western blots was analyzed further in Figs 6 and 7.

50 kDa molecular weight cut-off units (Ultra 0.5 ml centrifugal filters, Merck Millipore, Darmstadt, Germany) were used to separate serum from low molecular weight proteins and concentrate (10X) via centrifugation at 14000 rpm for 1 hr at 4°C, as summarized in Fig 1. Sera were stored at -80°C until tested. Prior to the start of immunoreactions, supernatants were collected and protein estimation was performed using Coomassie Plus Assay (Bradford method) reagent according to the manufacturer's protocol (Thermo Fisher Scientific, Rockford, IL).

### Rodents

8–10 weeks old Sprague-Dawley (SD) rats were housed in a controlled environment (21±1°C; humidity 60%; 12 hr-12 hr light-dark cycle; ad libitum access to food and water). Procedures involving animals and their care were conducted in conformity with the institutional guidelines that are in compliance with international laws and policies (EEC Council Directive 86/609, OJ L 358, 1, Dec.12, 1987; Guide for the Care and Use of Laboratory Animals, U.S. National Research Council, 1996). Cleveland Clinic Institutional Animal Care and Use Committee (IACUC) approved the protocol number 2015–1481 to perform the presented experiments. Brain sections and protein extracts from 6 SD male rats were used for the presented experiments.

### Immunohistochemistry

Rats were deeply anesthetized with isoflurane inhalation, followed by intracardiac perfusion using phosphate buffer solution (PBS, 1X). Brains were washed in PBS and glued with Scotch super glue to a metal plane, which in turn was immersed in ice-cold PBS before sectioning with a calibrated Vibratome 3000 (The Vibratome Co., St. Louis, MO) to generate 100 μm thick free-floating sections. The sections were collected in 6-well plates containing fresh PBS and placed on ice. Immunostaining for the identification of brain antigens was conducted using 3, 3'-diaminobenzidine (DAB). Contrary to the regular protocols, tissue sections were not permeabilized with detergents. Endogenous peroxidase was quenched with 0.3% hydrogen peroxide in methanol for 20 min. Further, blocking was done for 1 hr in blocking solution (2% goat serum in PBS) followed by overnight incubation with human serum (1:1000 in blocking solutions). Sections were then washed with PBS and incubated for 1 hr with a biotinylated anti-human IgG (1:600, BA-3000, polyclonal, Vector laboratories, Burlingame, CA) prepared in blocking solution. After PBS washes, sections were incubated for 1 hr with an avidin/biotin complex (Elite Vectastain ABC Kit, Vector laboratories, Burlingame, CA). This was followed again by PBS washes. The antibody binding sites were then visualized using DAB (Peroxidase Substrate Kit, Vector laboratories, Burlingame, CA). Finally, tissue sections were rinsed, dehydrated, cover-slipped with permount histological mounting medium (Fisher Scientific, Pittsburg, PA) and observed under bright-field microscopy (Leica Leitz, Leica Microsystems, Allendale, NJ).

### Immunofluorescence

Immunostaining for IgG (human), NeuN, GFAP, and CD31 in free-floating rat brain sections was performed as described below. Brains harvested from perfused SD rats were fixed in 4% paraformaldehyde (PFA) solution (in PBS) followed by cryopreservation in 30% sucrose (in PBS) at 4°C. 30 μm floating coronal sections were obtained using a cryostat (Leica CM3050, Leica Microsystems Inc, Buffalo Grove, IL), which were then washed in PBS followed by simultaneous permeabilization and blocking in blocking solution (3% goat serum and 0.3% Tween in PBS) for 30 min. Brain sections were then incubated in primary antibodies [human serum (IgG; 1:1000) and mouse anti-neuronal nuclei (NeuN; 1:500, MAB377, monoclonal, Chemicon, Darmstadt, Germany) or mouse anti-glial fibrillary acidic protein (GFAP; 1:100, G3893, monoclonal, Sigma, St. Louis, MO), mouse anti-cluster of differentiation 31 (CD31; 1:100, ab64543, monoclonal, abcam, Cambridge, MA)] followed by secondary antibodies [fluorescein anti-human IgG (1:1000, FI-3000, polyclonal, Vector laboratories, Burlingame, CA) and Alexa Fluor 594-conjugated affinipure donkey anti-mouse IgG (1:200, 715-585-150, polyclonal, Jackson ImmunoResearch Laboratories Inc., West Grove, PA) for overnight (4°C) and 2 hr (RT), respectively. Sections were then incubated in 0.1% Sudan black B to block auto-fluorescence, coverslipped in glass slides using Vectashield mounting medium with 4',6-diamidino-2-phenylindole (DAPI) (Vector laboratories, Burlingame, CA), and visualized by fluorescent microscopy (Leica Leitz, Leica Microsystems, Allendale, NJ). Fluorescence intensity and co-localization of cells and DAPI were measured by Q-Capture software (Q-Capture, Surrey, BC, Canada).

### Quantification of immunostaining

**Fig 2:**
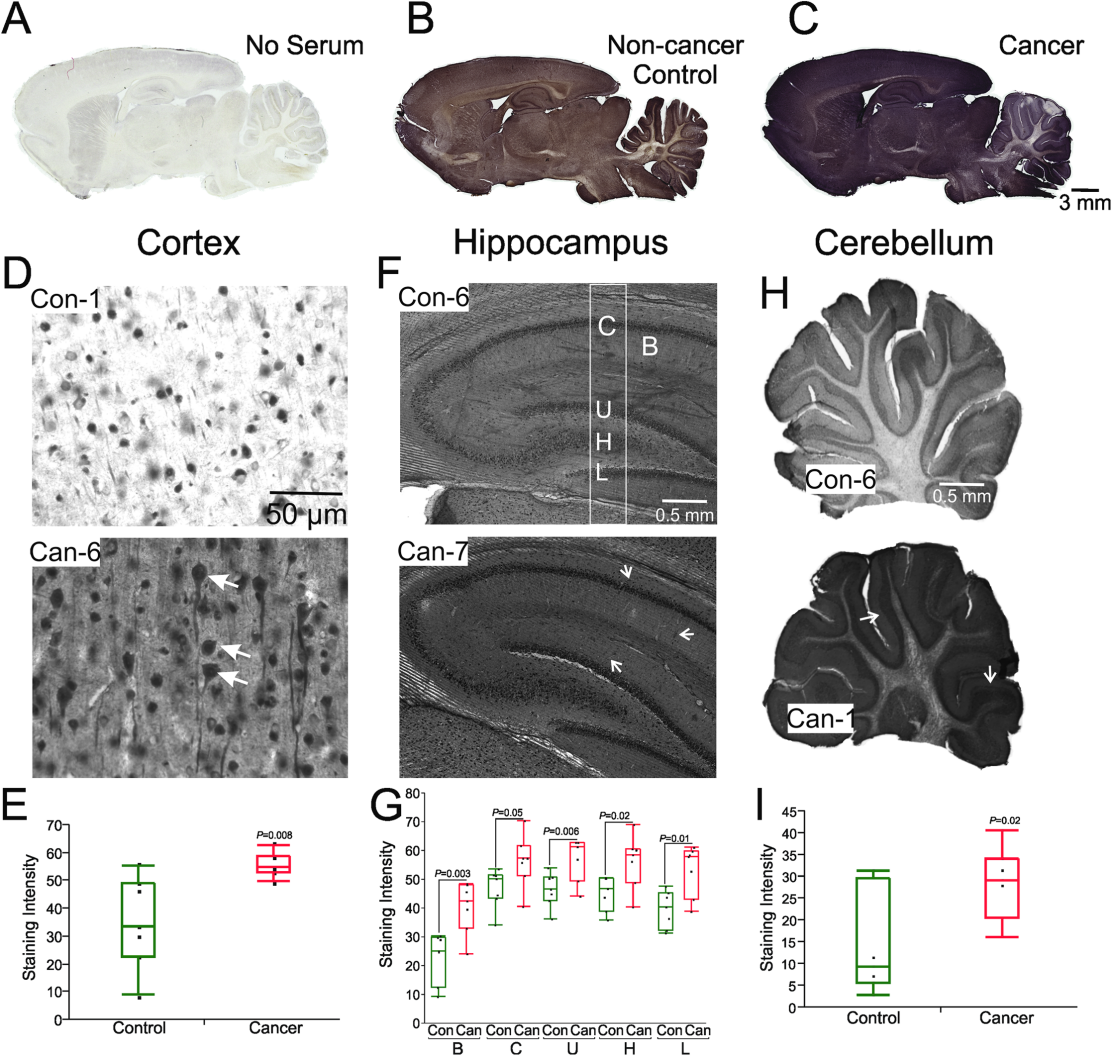
Sera from lung cancer patients are immunoreactive in rat brain. Sagittal brain sections were incubated with sera from cancer patients or non-cancer control subjects. A) No signal was detected in the absence of serum (negative control). B-C) Cancer-IgGs show robust immunoreactivity compared to control. Strong immunoreactivity was detected in the cortex, hippocampus, and cerebellum. D, F, H) Immunocytochemical staining with representative control (top row) and cancer (bottom row) sera. In cortex, staining intensity was significantly higher in cancer compared to control group (*P* = 0.008) (E). Autoantigen-directed immunoreactivity was observed in pyramidal neurons and their dendrites (arrows, D; also see Figs 3 and 4A). F-G) Brain sections incubated in cancer sera had higher immunohistochemical intensity in the hippocampal areas, Baseline (*B*; *P* = 0.003), CA1 (*C*; *P* = 0.05), upper blade of the dentate gyrus (*U*; *P* = 0.006), hilus (*H*; *P* = 0.02), and lower blade of the dentate gyrus (*L; P* = 0.01). Areas selected for intensity quantification are shown in F (top). Autoreactive IgGs were also detected in blood vessels (arrows point, F; also see Fig 4D). H-I) Overall staining intensity in the cerebellum was greater with serum IgGs from cancer patients than control subjects (*P* = 0.02). The granule cell layer was more prominently stained with sera from cancer patients as pointed in a representative image (H, lower panel). The average difference in the immunoreactivity signals between cancer and control sera in the cortex, hippocampus, and cerebellum were 20.6, 17, and 15.1 arbitrary units, respectively. Con, control; Can, cancer; Data are shown as mean ± SEM for 7 patients.

In order to compare multiple specimens, staining procedures (reagents, tissue processing), image acquisition settings, and exposure times were kept identical for the entire set. The quantification of DAB immunohistochemical signals was performed by ImageJ software as previously described [20], where a threshold was defined and kept identical throughout the analysis. The scoring method used is as described previously [21] and the scanned brain sections were scored by a blinded reviewer to avoid bias interpretation. In brief, the staining intensity was expressed in arbitrary units. Staining intensity analyses of cortex (20X magnification), hippocampus (1.5X magnification), and cerebellum (1.5X magnification) were performed in three randomly selected images for individual subjects (n = 7 for each group). Each dot in Fig 2E, 2G and 2I represents an averaged intensity for each serum. In the hippocampus, a rectangular area was drawn as shown in Fig 2F that automatically provided staining intensities of dark areas namely, CA1(C), hilus (H), and the upper (U) and lower (L) blades of the septal dentate gyrus. Fluoresced cells and nuclei were counted directly by marking colors in the images of 40X magnification.

### Western blotting

PBS-perfused rat brains were dissected into cortex, hippocampus, and cerebellum. Tissue lysates from these regions were extracted in radioimmunoprecipitation assay (RIPA, 1X) lysis buffer (Sigma-Aldrich, St. Louis, MO) containing protease inhibitors (Aprotinin 2 μg/ml, Leupeptin 5 μg/ml, Pepstatin 1 μg/ml, and PMSF 1 mM). Protein concentration was measured using Coomassie Plus Assay (Bradford method) reagent according to the manufacturer's protocol (Thermo Fisher Scientific, Rockford, IL). 50 μg proteins were then separated using 10% SDS-PAGE gels and electrotransferred onto polyvinylidene fluoride (PVDF) membranes. Equivalent sample loading and transfer was confirmed by Ponceau S staining. Membranes were then probed overnight with human serum (1:5000) followed by incubation with HRP-conjugated secondary goat anti-human (1:5000, 401445, polyclonal Calbiochem, Darmstadt, Germany) antibody. Protein bands were visualized using Pierce enhanced chemiluminescence (ECL) Western Blotting Substrate (Thermo Fisher Scientific, Rockford, IL). Kodak X-ray film developers were used to develop the X-ray films in the dark room. The densitometry analysis of bands was performed using ImageJ software following the standard protocol provided by National Institutes of Health (NIH).

### Statistical analysis

Data are expressed as mean ± standard deviation. Statistical significance was determined by either a Student’s t-test, one-way analysis of variance (ANOVA) or Mann-Whitney-Wilcoxon test using JMP 10 or OriginPro 9.0. The Bonferroni's or Dunnett's multiple comparison post-hoc tests were used as appropriate. The latter statistical analysis used is justified considering the relatively low ‘n’; however encompassing the appropriate analysis used for the interpretation of the presented data to compare the two major subject category in the current study (Control = 18 and Cancer = 18). Significance level of P < 0.05 was used for hypothesis testing.

### Epitope identification

The human and rat amino acid sequences of potential autoantibody targets listed in S3 Table were compared by a sequence comparison algorithm, BLASTp. Details on accession numbers and sequence comparison results are provided in S2 Table. The immune epitope database and analysis resource (IEDB; http://www.iedb.org) was used to search human epitopes involved in autoimmunity [22]. The entire amino acid sequence (human protein) was entered in IEDB.org to search linear peptidic epitopes homologous to the input sequence with at least 80% identity. Search results of 8–28 amino acid sequences were then located in the human protein and challenged for the presence of homologous and corresponding sequence in rat. A list of target antigens with homologous epitopes is provided in S3 Table.

## Results

We examined the antigenic reactivity of serum samples obtained from 18 NSCLC patients (66.6% male, 55.6% adenocarcinoma, 94% former smoker, 39% COPD, 17% diabetic mellitus) and 18 non-cancer control subjects (55.6% male, 67% former smoker, 11% COPD, 6% diabetic mellitus). Autoantigen reactivity was tested on rat brain sections or denatured protein extracts. NSCLC patients were diagnosed at stages I-IV. Table 1 summarizes the major clinical features, age, lifestyle, and habits of study subjects.

**Fig 3:**
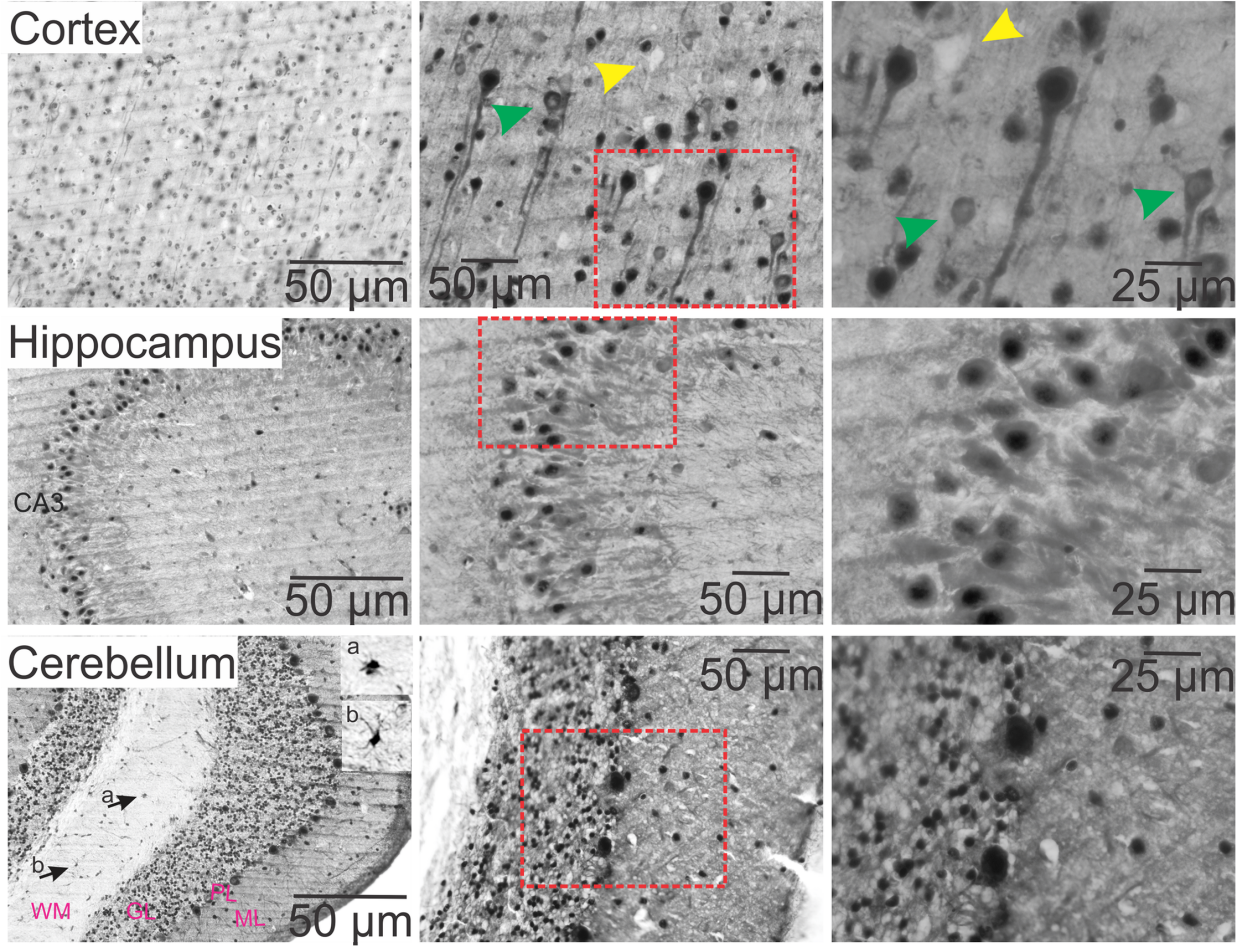
Characterization of cancer-IgGs binding against rat tissue in cortex, hippocampus, and cerebellum. The top panel shows a section from cortex stained with serum from a cancer patient. Autoreactive antibodies targeted cell plasma membrane, cell bodies, and axons/dendrites. The nucleus was not uniformly stained and a few scattered cells were devoid of nuclear staining (green arrowheads). A population of neurons was not stained at all (opaque area, yellow arrowheads). The middle panel shows a CA3 hippocampal region where, similar to the cortex, immunoreactivity was found in the nucleus and cell body. The bottom panel shows a cerebellar section where immunoreactivity was highest in the Purkinje and granular cell layers. However, some cells in the white matter (a & b, arrows point and insets) and molecular layer were also stained. Tissue sections were stained with Can-2 serum. WM, white matter; GL, granular layer; PL, Purkinje cell layer; ML, molecular layer.

**Fig 4:**
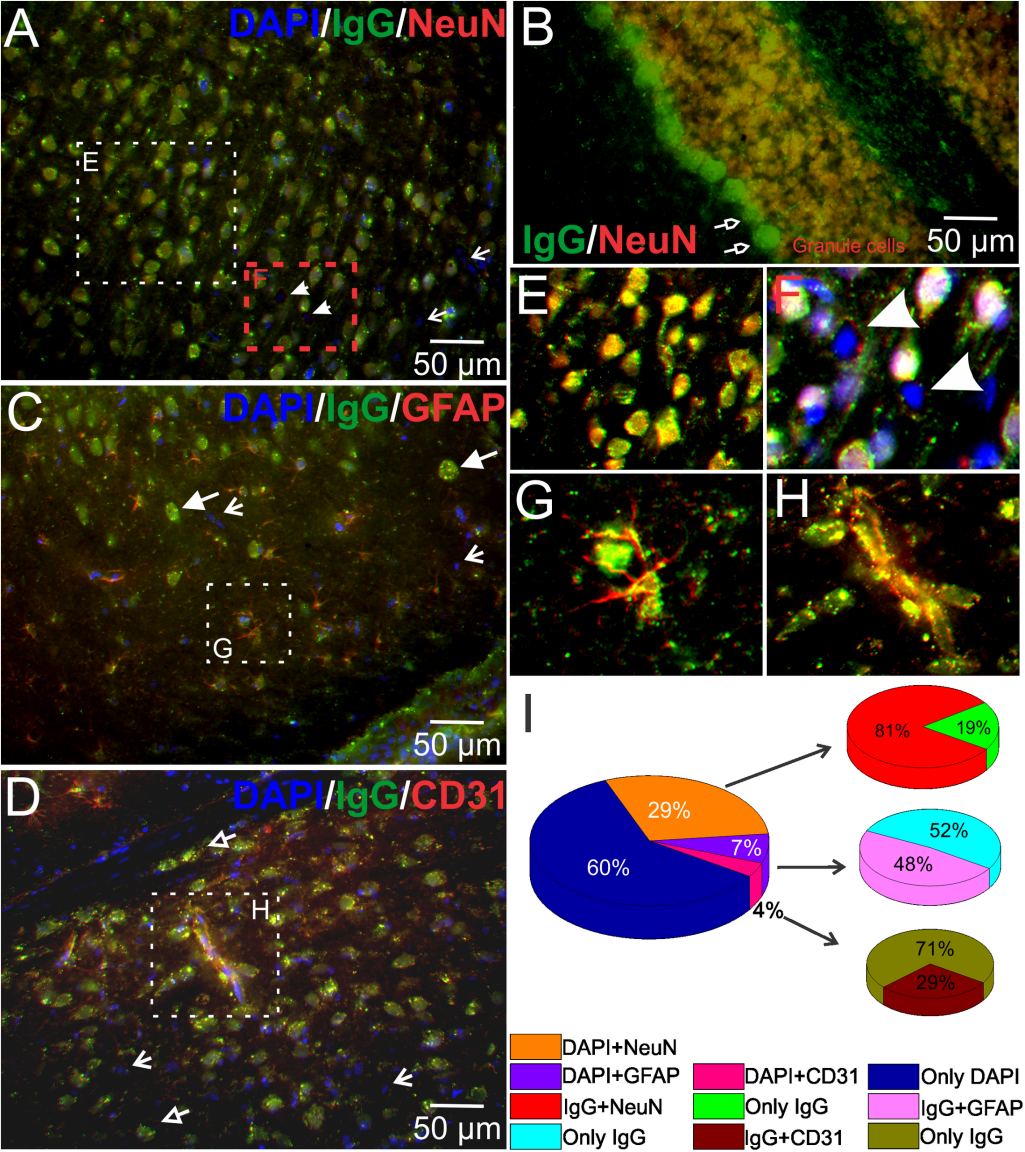
Autoantibodies are primarily directed against neuronal antigens. The figures show co-localization of cancer-derived autoreactive immunoglobulins (green) and neuronal (NeuN, red), astrocytic (GFAP, red), and endothelial (CD31, red) markers. Immunoreactivity for serum IgGs demonstrated staining of most cortical (A) and cerebellar (B) neurons. Small areas of ‘A’ were expanded in ‘E’ (cancer-IgG and anti-NeuN) and ‘F’. Some neurons were neither positive for cancer-IgG nor for anti-NeuN antibody (F, arrowheads). Purkinje cells were negative for NeuN (B, open arrows), nonetheless both Purkinje and granule cells immunoreacted with sera IgGs (B). A few IgG positive cells were defined as astrocytes (C) and endothelial cells (D). Small areas of ‘C’ and ‘D’ without DAPI staining were magnified to ‘G’ and ‘H’, respectively. The content of IgG in astrocytes was limited to the cytosol and to a few processes. The majority of cells in these panels were positive for cancer-IgGs (C, triangle arrows point; D, open triangle arrows point; green). A small percentage of cells remained unstained for IgGs (A, C, & D, simple arrows). I) Quantification of IgGs and cell-specific proteins in cortex. 81% of neurons were co-stained with cancer-derived IgG while 48% and 29% of astrocytes (by GFAP) and endothelial cells (CD31) were co-localized with cancer-IgG, respectively. NeuN, neuronal nuclei; GFAP, glial fibrillary acidic protein; CD31, cluster of differentiation 31.

Seven serum samples were randomly selected from each patient population to evaluate immunoreactivity of the sera on specific brain regions in freshly isolated and unfixed brain sections, keeping identical incubation time. In order to keep cell membranes intact, no detergent was added to the staining buffer. Fig 2 shows a typical outcome of these experiments. The scoring method used as previously described [21]. Scanned brain sections were scored by blinded reviewers from 0 (no visible staining) to 5 (intense staining). The reviewers performed all the scoring consistently across the brain slices and were unaware of specimen details. Three patients in the cancer group shared a score 5 and one had a score of 2, whereas the highest score for the control group was 2, reported for four patients. The overall immunoreactivity was higher in cancer compared to control, which was analyzed further in higher magnification to pinpoint the exact topographic localization of autoreactive immunoglobulins (Figs 2–4). Sections incubated in staining buffer without serum did not show any detectable signal (Fig 2A). However, even at the lower magnification, intense immunoreactivity was seen in cortex and hippocampus (Fig 2B vs 2C). A detailed chromogenic immunohistochemical analysis was performed for cortex, hippocampus, and cerebellum (Fig 3).

The average difference in the immunoreactivity signals between cancer and control sera are summarized in Fig 2. These results suggest no specific regional targeting by autoimmune IgGs.

The distribution of immunoreactivity in the cortex is shown by representative images for each group in Fig 2D. The overall staining intensity was stronger in all cortical regions stained with sera from cancer patients (P = 0.008, Fig 2E). Even at lower magnification, medium-large pyramidal and cylindrical-shaped neuronal cells were heavily immunoreactive, as pointed in Fig 2D (lower panel, arrowheads). This self-directed immunoreactivity was most strongly associated with antigen in the nucleus, cell body, and axons of most of these neurons (also see Fig 3). Interestingly, immunoreactivity appeared to target mainly the plasma membrane of some neurons, as indicated in Fig 3 (green arrowheads). Scattered unstained neurons were detected in the cortex; this, however, was not consistently seen (compare Fig 2D, Can-6 and Fig 3, Can-2; yellow arrowheads).

In the hippocampus, self-reactive IgG signal was confined largely to the pyramidal cell layers and dentate gyrus. There was comparably less staining of the molecular layer of the dentate gyrus, strata lacunosum moleculare, radiatum, and oriens (Fig 2F). We quantified staining intensities of the hippocampal cell layers as described in the Method section. As shown in Fig 2G, staining intensity was significantly higher in cancer at CA1 (C, P = 0.05), upper blade of the dentate gyrus (DG) (U, P = 0.006), hilus (H, P = 0.02), and lower blade of the DG (L, P = 0.01). Cellular analysis showed that the principal cells (CA1-CA3 and hilar cells) and granular neurons were heavily stained by IgGs. In the CA3 pyramidal cell layer, pyramidal cell bodies, apical dendrites, and nuclei were stained; the latter had the highest staining intensity (Figs 2F and 3, middle row). Immunoreactivity was also detected in the blood vessels (Fig 2F, lower panel, arrow points; see also Fig 4D).

In the cerebellum, autoreactive IgGs were localized mainly in the Purkinje and granular cell layers (Figs 2H and 3). The overall staining intensity was significantly higher (P = 0.02) in cancer than control group (Fig 2I). Though IgGs were mostly found in the cell bodies and nuclei of Purkinje cells and the granular cell layers, there was no obvious staining of the dendritic spines of the Purkinje cells or fibers arising from granule cells (Fig 3, bottom row). However, axons in white matter neurons were stained by autoimmune IgGs (Fig 3, bottom row, arrow points and insets).

Fluorescence co-localization analysis was performed to identify serum IgG binding to specific cell types. Fig 4 shows a typical outcome of experiments where frozen rat brain sections were stained for the presence of neuronal, astrocytic, and endothelial cell markers. In cortex, IgGs were found in most neurons (Fig 4A and 4E). Quantitative analysis of immunofluorescence showed that 81% of IgG-positive cells were co-localized with anti-NeuN, where 19% stained with only autoreactive IgGs (Fig 4I). Hippocampal pyramidal cells were also co-stained by autoreactive IgGs and anti-NeuN antibodies. Anti-NeuN has been utilized extensively in histological analyses to stain most neuronal cell types with only a few exceptions, e.g., cerebellar Purkinje cells [23]. Thus, as expected, serum IgGs and anti-NeuN antibodies were co-localized in granular cells of the cerebellum, but not in the Purkinje cells (Fig 4B, open arrows point). However, both of these neuronal cell types were positive for autoreactive IgG staining (green). We detected a small population of cortical neurons that were neither stained by autoreactive IgGs nor by anti-NeuN-specific antibodies (Fig 4F, arrowheads; also see Fig 3, top row). These results also show the co-localization of IgG and DAPI in astrocytes (Fig 4C, GFAP, red) and endothelial cells (Fig 4D, CD31, red). Quantitative analysis of autoreactive immunofluorescence in the cortex showed 7% GFAP positive astrocytes, while 4% were positive for CD31 (endothelium). Furthermore, among astrocytes, almost half (48%) contained autoreactive IgGs, whereas only 29% of IgG-stained cells were endothelial cells (Fig 4I). Localization of IgGs in astrocytes and endothelial cells were also detected in the hilus, stratum lacunosum-moleculare, radiatum, and oriens of the hippocampus. Within three staining groups, 60% of the cells were neither stained with IgG nor with cell identity markers (Fig 4I).

**Fig 5:**
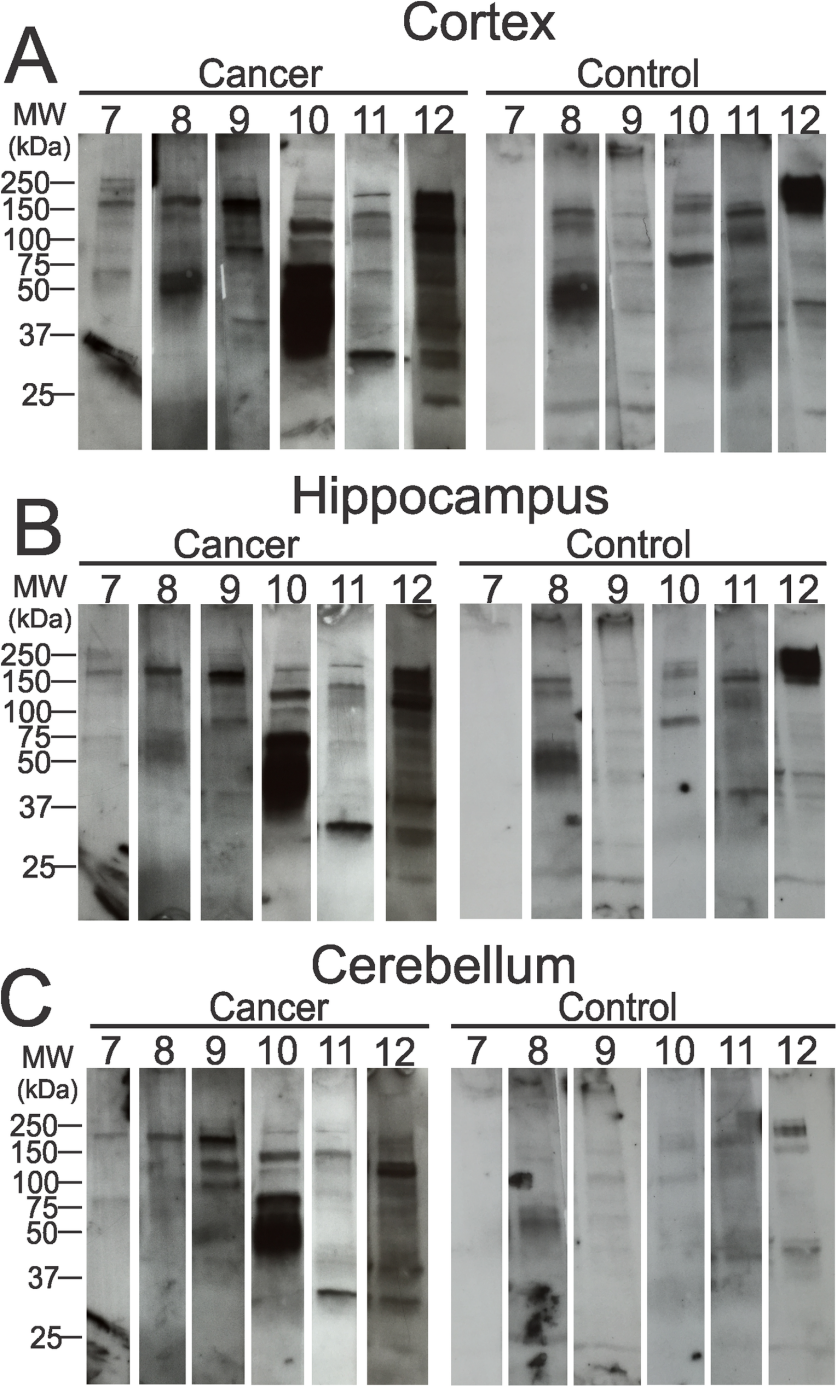
Immunoblots of rat brain extract probed with serum immunoglobulins. Cortex (A), hippocampus (B), and cerebellum (C) protein extracts were separated by SDS-PAGE and blotted with serum IgGs collected from cancer patients or non-cancer control subjects to determine the immunoreactivity at specific molecular weights. Representative blots show reactivity of 6 subjects in each group. The position of bands and their intensities show serum IgG autoantibody profiles are unique to individual subject.

In order to narrow down the antigenic targets of autoreactive IgGs, protein extracts isolated from cortex, hippocampus, and cerebellum were fractionated by SDS-PAGE and analyzed by Western blotting. Immunoreactivity bands developed by probing sera of cancer and control subjects (6 out of 18) are shown in Fig 5A–5C. The band intensities were distinctly greater for the cancer group. However, IgG autoantibody profiles were found to be variable even within each group. The prevalence of individual bands at 25–250 kDa in the cancer and control patients is shown in Table 2. The most prevalent band in cancer, in order of frequency, was the 130 kDa band. In cortex and hippocampus (P = 0.05), this band was positive in 7/18 (39%) of control subjects (6/16, 38% cerebellum) and 13/18 (72%) of cancer patients (10/16, 63% cerebellum; P = 0.2). Higher molecular weight bands, particularly 250 and 150 kDa, were recognized by at least 9/18 (50%) subjects regardless of cancer.

**Table 2:** Prevalence of individual bands on Western blot in cancer patients and non-cancer control individuals.

Brain regions	Subjects	Molecular weight (kDa)													
		250	200	150	130	100	85	75	65	50	45	40	37	30	25
Cortex	Control (N = 18)	14	8	9	7	6	6	11	4	11	1	7	3	8	8
	Cancer (N = 18)	14	10	10	13	10	3	5	7	9	4	5	5	7	5
	P value	1.0	0.5	0.7	0.05*	0.2	0.3	0.05*	0.5	0.2	0.5	0.4	0.8	0.8	0.3
Hippocampus	Control (N = 18)	13	6	9	7	4	5	10	1	10	2	4	2	6	8
	Cancer (N = 18)	13	9	11	13	9	2	7	4	9	4	5	5	6	5
	P value	1.0	0.3	0.5	0.05*	0.1	0.2	0.3	0.2	0.8	0.4	0.7	0.2	1.0	0.3
Cerebellum	Control (N = 16)	8	3	9	6	1	4	9	1	8	5	1	2	3	8
	Cancer (N = 16)	9	7	9	10	9	1	5	2	6	3	4	2	7	3
	P value	0.7	0.1	1.0	0.2	0.003*	0.2	0.2	0.6	0.5	0.4	0.2	1.0	0.2	0.07

* Statistically significant

The number of autoimmune bands in lung cancer patients and control individuals is shown in Table 3. In cortex, the average number of bands was 5.9 (lowest = 2, highest = 10) for cancer and 5.7 (lowest = 0, highest = 9) for control. This pattern was similar to the number of bands counted in hippocampal (cancer, average = 5.7, lowest = 2, highest = 10; control, average = 4.9, lowest = 0, highest = 8) and cerebellar protein extracts (cancer, average = 4.8, lowest = 1, highest = 10; control, average = 4.3, lowest = 0, highest = 9). Even though the numbers of bands between two groups were similar, we measured differences in the level of individual band intensities (S1 Fig). The majority of the positive bands in samples from control individuals were comparably faint. In cortex, hippocampus, and cerebellum, the average band intensity was greater in cancer patients at 30, 37, 45, 50, 65, and 100 kDa than controls (S1A Fig). Moreover, combined average intensities of all three regions also have similar trends. We also plotted the total intensity of cancer and control groups at 25–250 kDa (S1B Fig). The highest elevation was detected at 30, 37, 45, 65, and 100 kDa. The fold differences in cancer compared to control are provided in S1B Fig.

**Table 3:** Number of bands on Western blot in lung cancer and non-cancer control subjects.

Brain regions	No. of positive bands	Cancer patients (N = 18)	Control patients (N = 18)
Cortex	0	0	1
	1–3	2	2
	4–6	7	7
	7–9	8	8
	>9	1	0
Hippocampus	0	0	1
	1–3	4	3
	4–6	7	10
	7–9	6	4
	>9	1	0
Cerebellum	0	0	1
	1–3	5	4
	4–6	10	10
	7–9	2	3
	>9	1	0

**Fig 6:**
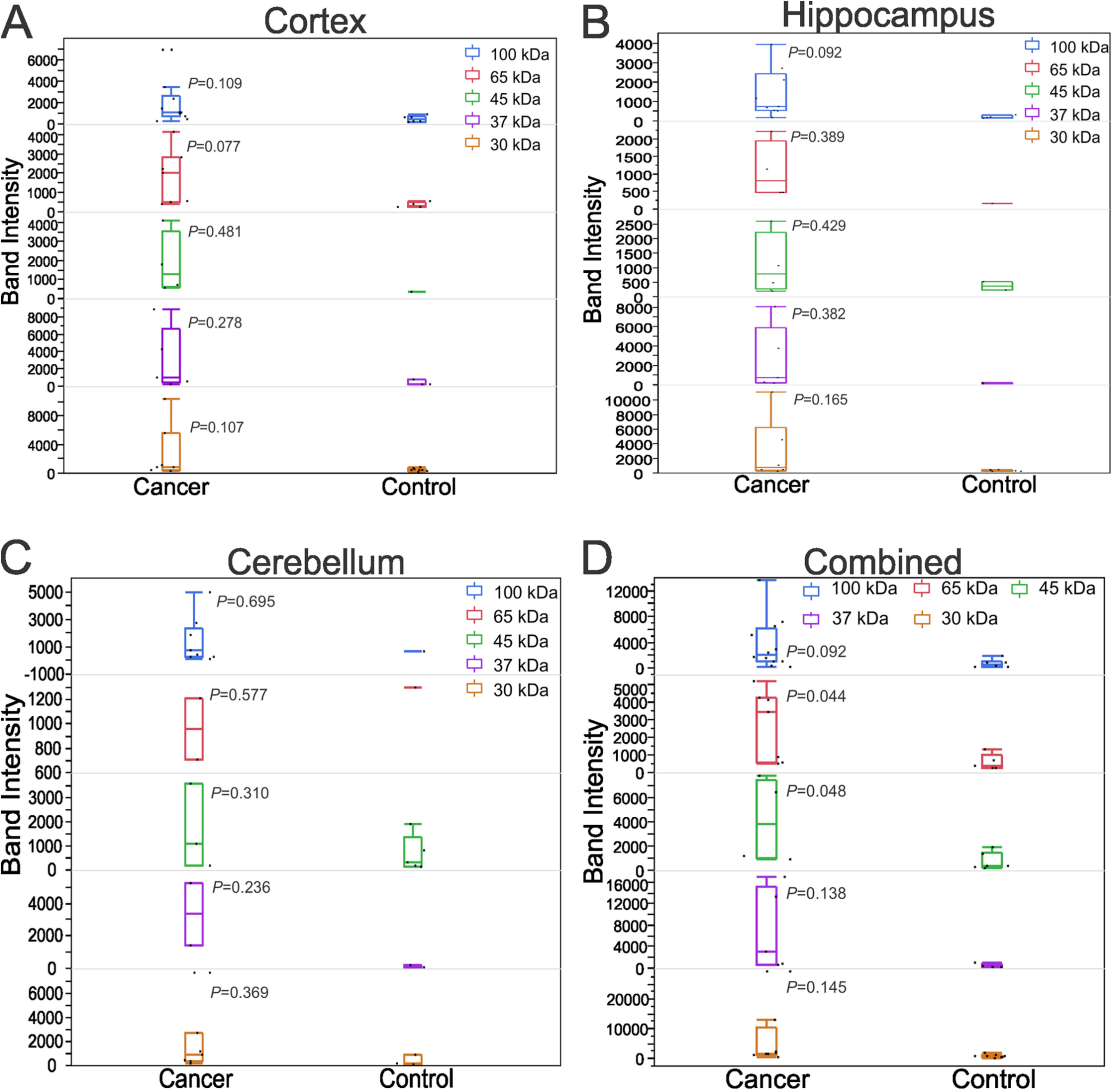
Western blot band intensity is higher for serum samples from cancer patients. The average band intensity was greater in cancer group at 30, 37, 45, 65, and 100 kDa. This pattern was similar in cortex (A), hippocampus (B), and cerebellum (C). In addition, the pooled intensity from these regions was significantly increased in cancer relative to control at 65 (*P* = 0.044) and 45 kDa (*P* = 0.048) (D).

The five molecular families with the highest differences in band intensity compared to control were chosen for further analysis. The data were expressed as mean ± SD for each group with respective P-value (relative to control) (Fig 6). The average intensity was higher in cancer, although this was not statistically significant. In addition, a much smaller standard deviation was observed in the control group for molecular weights (30, 37, 45, 65, and 100 kDa). We have ascertained similar patterns in the protein extract isolated from cortex (Fig 6A), hippocampus (Fig 6B), and cerebellum (Fig 6C). The results obtained by combining band intensities of all three regions followed the same pattern (Fig 6D). Also, the immunoreactivity intensity was significantly higher at 45 (P = 0.048) and 65 kDa (P = 0.044).

**Fig 7:**
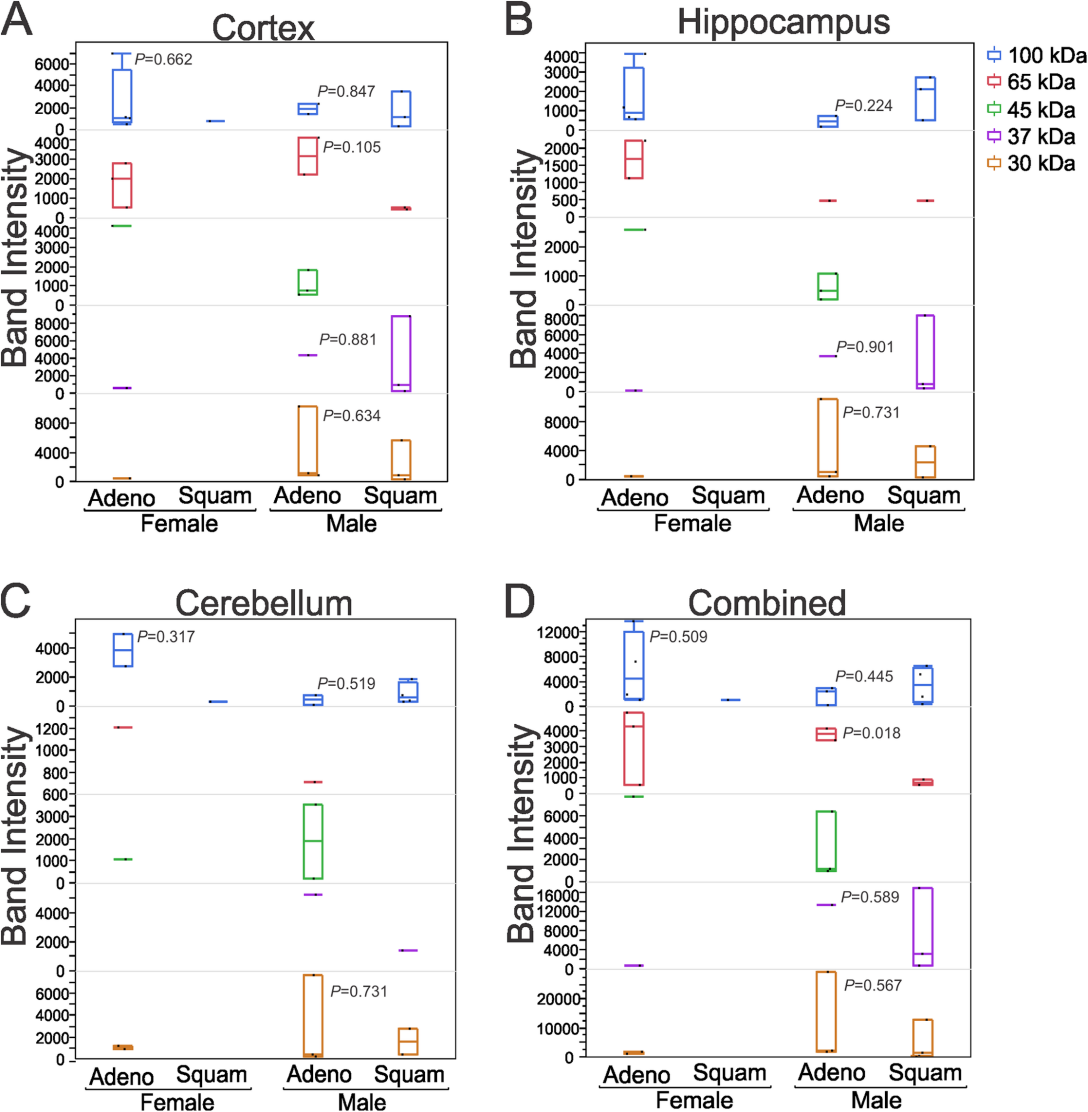
A family of 100 kDa proteins is a primary target of serum IgGs from female lung adenocarcinoma patient. Gender- and disease-dependent (adeno vs squamous cell carcinomas) signals at 30, 37, 45, 65, and 100 kDa. Four of 5 female adenocarcinoma patients’ serum immunoglobulins detected an immunoblot band at 100 kDa in the cortical (A) and hippocampal (B) protein extracts. In cerebellar protein extracts, the maximum number of band detected was 4 (out of 7) at 100 kDa by the serum of male squamous cell carcinoma patient (C), which was the highest among all three regions. In the case of sera from male patients, proteins recognized by IgGs were variable in both adeno- and squamous carcinoma group. D) The pooled intensity from all three regions showed significant difference in adenocarcinoma male (*P* = 0.018, compared to squamous male). *P* values relative to squamous are provided in the graph. Adeno, adenocarcinoma; squam, squamous cell carcinoma.

To test the differential prevalence of brain reactive autoantibodies in adenocarcinoma or squamous cell carcinoma among male and female patients, we further compared 30, 37, 45, 50, 65, and 100 kDa Western blot bands (Fig 7). In men, squamous cell carcinoma was the dominant histological type (58.3%), while adenocarcinoma was more frequent among female patients (83.3%). Four of five (80%) female patients with adenocarcinoma demonstrated specific reactivity for a 100 kDa antigen band (Fig 7A and 7B). However, only 2 out of 5 were reactive against 100 kDa cerebellar protein (Fig 7C). Interestingly, antibodies directed against low molecular weight proteins (30, 37, and 45 kDa) were less frequent (1/5 i.e., 20% in cortex, hippocampus, and cerebellum) or absent (37 kDa, cerebellum) in female adenocarcinoma patients. On the other hand, 3 out of 5 (60%) male patients with adenocarcinoma demonstrated reactivity at 30 kDa against protein in rat cortex, hippocampus, and cerebellum (Fig 7A–7C). The frequency of hippocampal and cerebellar protein detection was also 3 out of 5 (60%) at 45 kDa in these patients. 3/7 (42.8%, highest frequency) male squamous cell carcinoma patients showed specific reactivity at 30, 37, and 100 kDa against proteins in the cortex (37 and 100 kDa against hippocampal protein). In cerebellum, maximum reactivity (4/7, 57.1%) was measured at 100 kDa, whereas there was no reactivity at 45 or 65 kDa (Fig 7C). Similar patterns were observed in the graphs plotted after combining band intensities measured in cortical, hippocampal, and cerebellar protein extracts (Fig 7D). The frequency and intensity of protein detection at 100 kDa by female adenocarcinoma patients’ antibodies are highly evident. However, in male patients protein detection was much more heterogeneous among adenocarcinoma or squamous cell carcinoma groups. The identity of the proteins at 100, 65, 45, 37, and 30 kDa are currently unknown, however a list of possible targets is provided in S1 Table.

The amino acid sequences of potential targets listed in S1 Table were compared for sequence identity between human and rat protein (S2 Table). As an example, the glutamate receptor GluR2 had the lowest (32%) while SOX transcription factor 1 (SOX-1) and protein kinase gamma (PKCγ) had the highest (99%) sequence similarity. Of these protein candidates, only those sharing more than 87% linear epitope identity were possible to locate in human and rat sequences using IEDB (S3 Table). These epitopes ranged from 9 to 28 amino acids. In glutamic acid decarboxylase 2 (GAD2), HuA, α-enolase, aquaporin 4 (AQP-4), and protein tyrosine phosphatase receptor type N (PTPRN), multiple distinct epitopes were identified in the same target protein. Some of the amino acid residues in human epitopes were different in rat (S3 Table; see highlighted residues); however, side-chain polarity and charge were largely conserved. For example, valine (V) is a neutral and nonpolar amino acid in the epitope of cerebellar degeneration-related protein 2 (CDR2), which corresponded in rodents to another neutral and nonpolar amino acid, leucine (L). A comprehensive list of target proteins with their epitopes is provided in S3 Table.

## Discussion

The main finding of this research is the presence of brain-directed antibodies in the serum of the majority of NSCLC patients who did not present any other signs, symptoms or clinical diagnoses of a typical autoimmune disease. Autoantibodies were predominately directed against pyramidal neurons (cortex and hippocampus), Purkinje cells (cerebellum), and granule cells (hippocampus and cerebellum). In addition, autoreactive sera also detected glial and endothelial antigens. Nuclear antigens were a primary target, but antibodies also localized to the cell membrane and in the cytoplasm. 30, 37, 45, 65, and 100 kDa proteins were the major targets of autoantibodies, with 100 kDa being most frequent in female adenocarcinoma patients.

PNNS is a rare condition occurring in less than about 1% of cancer patients [24]. In most patients, neurological symptoms develop before the diagnosis of a tumor [25]. Autoimmune processes, triggered by the neoplasm, are directed against a common antigen present in the cancer and nervous system. Its pathogenesis is the aberrant expression of an antigen in the tumor that is normally expressed only in the nervous system. Even if tumor antigens are identical in structure to the neuronal antigens, they may be recognized as non-self, leading to an immune attack directed against both tumor and nervous system [26]. The discovery of onconeural antibodies has led to recognize this hypothesis [25]. However, the molecular targets and role of onconeural antibodies in the neurological dysfunction are still elusive.

### Brain-directed autoantibodies in NSCLC

Even though a large number of PNNS are strongly associated with SCLC (3–5%) [14], based on case reports or small series it can also be diagnosed in NSCLC patients [15–18]. We have for the first time characterized brain-directed autoantibodies in NSCLC patients (66% male, 41% adenocarcinoma; 83.3% female adenocarcinoma). Squamous cell carcinoma is less frequent in the female population of patients compared to adenocarcinoma [1], which is also reflected in our randomly selected group of NSCLC patients. We found that autoreactive serum IgGs in 70% of cancer patients are more reactive against brain tissue compared to sera from control subjects. This supports the notion that brain-directed antibodies can be found also in NSCLC patients. The background staining was likely due to non-specific antibody binding to endogenous Fc receptors or a combination of hydrophobic and ionic interactions. Further, the greater staining intensity in some control sera treated brain sections were potentially due to pre-existing conditions that increase total antibodies such as infection and/or vaccination. It may be important to note that unlike in most other studies our ‘control’ population shared risk factors and clinical history/demographics with the NSCLC patients. It is therefore possible that autoantibodies are, in some of these subjects, present before a definitive diagnosis of lung cancer was made.

### Specificity of autoantibodies against different brain regions and cell types

We carried out a detailed analysis to separate systemic antibodies in NSCLC subjects based on regional specificity of CNS targets. The staining intensity was nearly identical in cortex, hippocampus, and cerebellum, suggesting antibodies have no regional preference for binding. This is not surprising, since endogenous autoantigen are likely expressed in multiple brain regions. For example, protein Hu involved in cerebellar degeneration, encephalomyelitis, and limbic encephalitis is expressed by cerebellar, hippocampal, and cortical cells [27, 28]. In addition, pyramidal cells are present as a major cell type in the cortex and hippocampus [29] and granule cells are present in both hippocampus and cerebellum [30].

Our study identified that neurons, astrocytes, and endothelial cells are the primary targets of autoantibodies, though autoantibody against oligodendrocytes was also measured in an autoimmune condition [31]. Among them, neurons are the major targets. Purkinje and granule cells in the cerebellum, pyramidal and granule cells in the hippocampus, and pyramidal cells in the cortex were the major neuronal types recognized by serum IgGs. Interestingly, a specific neuronal population was not revealed by autoreactive IgGs, suggesting heterogeneity of autoantibodies against the nervous system in NSCLC patients. Many studies have identified several categories of neuronal autoantibody targets, including nuclear or cytoplasmic antigens and cell-surface proteins (see review on neuronal autoantigens, [32]). However, we are among the first to report astrocytes and endothelial cells as the targets of autoantibodies in cancer patients.

### Identity of putative autoantigen in NSCLC

NMDAR is a neuronal glutamate-gated cation channel present in the synaptic membrane regions [33]. Systemic antibodies against NMDAR are commonly found in cancer patients [34]. However, recent studies provided definitive evidences for non-neuronal expression of the NR1 subunit of NMDAR, which pointed to astrocytes [35] and endothelial cells [36]. Based on above evidences, a pleiotropic targeting of several cell types by anti-NMDAR IgGs is not surprising. Our results support the presence of anti-NMDAR IgGs in NSCLC patients and their targeting of neuronal, glial and endothelial cells.

The microscopic examination of cells showed that IgG reactivity is present in different structures of brain but it’s most evident in the nuclei, axons, astrocytes processes, and endothelial membranes. Previously, we have detected nuclear antigens (histone complexes) as a major target of serum IgGs from patients with epilepsy [21]. These results are also supported by previous studies, which suggest that autoantibody targets can be nuclear (e.g., Hu, Ri, and Ma), cytosolic (e.g., CRMP5 and amphiphysin) or cell membrane (e.g., NMDAR, AChRs, and GABA_B_R) proteins [24]. It is important to note that nuclear and cytoplasmic antigens were detected by serum IgGs, even in the absence of a detergent permeabilization step. As demonstrated by Heffer-Lauc et al., permeabilization is not essential for nuclear and cytoplasmic binding in the immunohistochemistry assay, very likely because cells were damaged enough during the tissue preparation and staining steps [37].

### Molecular weight of autoantigen hints to broad array of targets in NSCLC

In addition to an immunocytochemical analysis, we performed an elaborate Western blot analysis of the autoantibody response against cortical, hippocampal, and cerebellar protein extracts. Cancer serum IgGs detected a large number of protein bands of various molecular weights. The presence of multiple antibodies for a specific neurological syndrome is common in PNNS. For example, anti-Hu, GAD2, NMDAR, and AMPAR are commonly associated antibodies in the limbic encephalitis [27, 28, 34, 38, 39]. These observations further support that PNNS is a heterogeneous neurological syndrome. The staining of autoantigen bands in the NSCLC patients was considerably more intense than control subjects, though there was no obvious difference in the frequency of band occurrence. This suggests that a low level of background signal is present in all sera. The inconsistency in autoantibodies among cancer patients is potentially due to demographics, tumor histology, lifestyle, previous disease conditions, habits, etc.

There were 5 notably prevalent, distinct bands with reproducibly defined positions of significant intensity in NSCLC sera at 30, 37, 45, 65, and 100 kDa. This result demonstrated the presence of large number of antibodies in the serum of NSCLC patients that are directed against antigens in the nervous system. In addition, these bands in the Western blots can be considered as marker bands to diagnose the prevalence of onconeural autoantibodies in NSCLC patients. The Center for Disease Control and Prevention recommends serological testing by ELISA followed by confirmatory test with Western blots to diagnose Lyme disease [40]. Similarly Western blot testing might be superior to existing ELISA or fluorescent staining techniques for the diagnosis of anti-brain antibodies. A list of potential antigen targets at these molecular weights is provided in S1 Table. The identification of conserved autoimmune epitopes in these human and rat antigens provide a molecular basis for the reactivity of human antibodies against rat proteins [22]. Further, our study indicates that an antibody titer is directly related to the number of epitopes. For example, multiple epitopes have been identified in Hu protein and anti-Hu antibodies are predominant in limbic encephalitis and lung cancer [41–44].

The prevalence of antibodies and their types are extremely diverse among cancer patients. For example, in a study of 200 SCLC patients, the prevalence rates of onconeural antibodies for Hu, CRMP5, amphiphysin, Ri, Ma2, and Yo were 22.5%, 0.5%, 2.5%, 1.5%, 1%, and 0.5%, respectively [11]. In our study of NSCLC patients, antibodies against 100 kDa band have highest specificity and sensitivity to female adenocarcinoma patients. However, antibodies against brain antigens in male adenocarcinoma and squamous cell carcinoma patients were less specific. The confirmation of this will require the analysis of larger and much more homogenous population. Nevertheless, these observations raise important questions regarding the potential role of autoantibodies in brain pathogenesis and/or in cancer progression.

### Clinical significance of autoreactive IgGs in NSCLC

The clinical significance of antibodies directed against CNS antigens has not been entirely understood and remains controversial. Voltage-gated calcium channel (VGCC) antibody has been studied by injecting IgGs from Lambert-Eaton myasthenic syndrome (LEMS) patients into the laboratory animals, which showed electrophysiological abnormalities characteristic for LEMS [45]. In contrast, the role of intracellular antigens such as Hu, CV2, Ri, and Yo is not yet clear [46]. In addition, it is not certain whether these antibodies can cause cell lysis [47]. Moreover, immunization of guinea pig with Hu or Yo antigens induced serum antibodies, but not any neurological disease [48]. Some studies suggest that PNNS with high anti-Yo, Ma, or Hu antibodies are probably due to T-cell mediated destruction of neurons [49]. Thus, the large numbers of autoantibodies seem to be only a marker of autoimmunity in cancer, not a disease.

Studies so far consider that brain-reactive autoantibodies are pathogenic, though a proper correlation between serum titers and brain pathology is still lacking [50]. Autoantibodies might have three possible actions in the brain: 1) Systemic antibodies can passively transfer into the brain and induce apoptotic neuronal death, as described previously by Huerta et al. [51]; 2) Autoantibodies may act as antagonists, blocking essential pathways which lead to abnormal nervous system development and/or function. This may cause either gain or loss of function depending on the specific pathways and antigen; or, 3) they may facilitate tissue destruction through either cell-mediated or complement-mediated cytotoxicity. These scenarios could potentially result in the alteration of neuronal functions, density and/or distribution of receptors, and modulate the release of neurotransmitters and/or cytokines. Additional studies are essential to determine whether the (auto/onco) antibodies have significant effects on brain structure and/or function.

In conclusion, the present results give a detailed account of the cellular and subcellular targets of serum antibodies from NSCLC patients. We report that antibodies in the serum of NSCLC patients are more selective and sensitive against the brain proteins at 30, 37, 45, 65, and 100 kDa. Further studies are essential to identify the antigens and characterize the mechanisms leading to brain dysfunction. However, this study opens an avenue for researchers to characterize these antibodies and potentially develop them as biomarkers to detect NSCLC before the cancer symptoms are apparent.

## Supporting information

S1 Fig Comparison of Western blot band intensities. **A.** The average (mean ± SD) band intensities at 25–300 kDa molecular weights were plotted for cortex, hippocampus, and cerebellum individually and in combination (combined). Right panel is a portion of the graph (left, dotted rectangle) zoomed for a closer look at the crowded points at lower molecular weights. Selected molecular weights were plotted in Fig 6B. **B**. The sum of the band intensities measured at 25 to 250 kDa was plotted for cortex, hippocampus, cerebellum, and total (combined). Intensity folds compared to control are provided in the cancer bar of the graph. 30, 37, 45, 65, and 100 kDa had highest intensity compared to control group, and thus, were selected for further analysis (see Figs 6 & 7). Hippo., hippocampus; Cereblm., cerebellum; Comb., combined.
(DOC)

S1 Table Candidate serum autoantibody targets in brain. **NMDAR-NR1**: N-methyl-D-aspartate receptor-subunit NR1, **GABABR**: Gamma amino butyric acid B receptor, **AMPAR**: α-amino-3-hydroxy-5-methyl-4-isoxazolepropionic acid receptor, **GluR**: Glutamate receptor, **DPPX**: Dipeptidyl-peptidase-like protein 6, **mGLuR1**: metabotropic glutamate receptor 1, **DNER**: Delta/Notch-like EGF repeat, **LGI-1**: Leucin-rich glioma inactivated 1 protein, a protein associated with voltage-gated potassium channels (VGKC), **CRMP5**: Collapsin response-mediator protein 5, **PKCγ**: Protein kinase C gamma, **CDR2**: Cerebellar degeneration-related protein 2, **AChRs**: Nicotinic acetylcholine receptors, **GAD2**: Glutamic acid decarboxylase 2, **SYT1**: Synaptotagmin 1, **ZIC**: Zinc fingers of cerebellum, **CV2**: Crossveinless 2, **Hu**: a group of RNA-binding proteins (HuA-HuD), **PNMA1/2 (Ma)**: Pareneoplastic Ma Proteins, **AQP-4**: Aquaporin-4, **MBP**: Myelin basic protein, **SOX-1**: Sex determining region Y-like high mobility group box 1 protein, **PTPRN**: Protein tyrosine phosphatase receptor type N, **ANNA-1**: Anti-neuronal nuclear antibody type I, **PCA-1**: Purkinje cell autoantibodies, **Pre.**: Predicted molecular weight, **Obs.**: Observed molecular weight.
(DOC)

S2 Table Protein sequence comparison between human and rat autoantibody targets listed in S1 Table. (DOC)

S3 Table Identification of epitope(s) in human and rat target proteins listed in S1 Table. (DOC)
