# Supplementary material for: Detection of brain-directed autoantibodies in the serum of non-small cell lung cancer patients
Source: PLoS One. 2017 Jul 26;12(7):e0181409. doi: 10.1371/journal.pone.0181409 (PMC5528996; doi:10.1371/journal.pone.0181409)
Supplement: S1 Fig — A. The average (mean ± SD) band intensities at 25–300 kDa molecular weights were plotted for cortex, hippocampus, and cerebellum individually and in combination (combined). Right panel is a portion of the graph (left, dotted rectangle) zoomed for a closer look at the crowded points at lower molecular weights. Selected molecular weights were plotted in Fig 6B. B. The sum of the band intensities measured at 25 to 250 kDa was plotted for cortex, hippocampus, cerebellum, and total (combined). Intensity folds compared to control are provided in the cancer bar of the graph. 30, 37, 45, 65, and 100 kDa had highest intensity compared to control group, and thus, were selected for further analysis (see Figs 6 & 7). Hippo., hippocampus; Cereblm., cerebellum; Comb., combined. (DOC) [file pone.0181409.s001.doc]

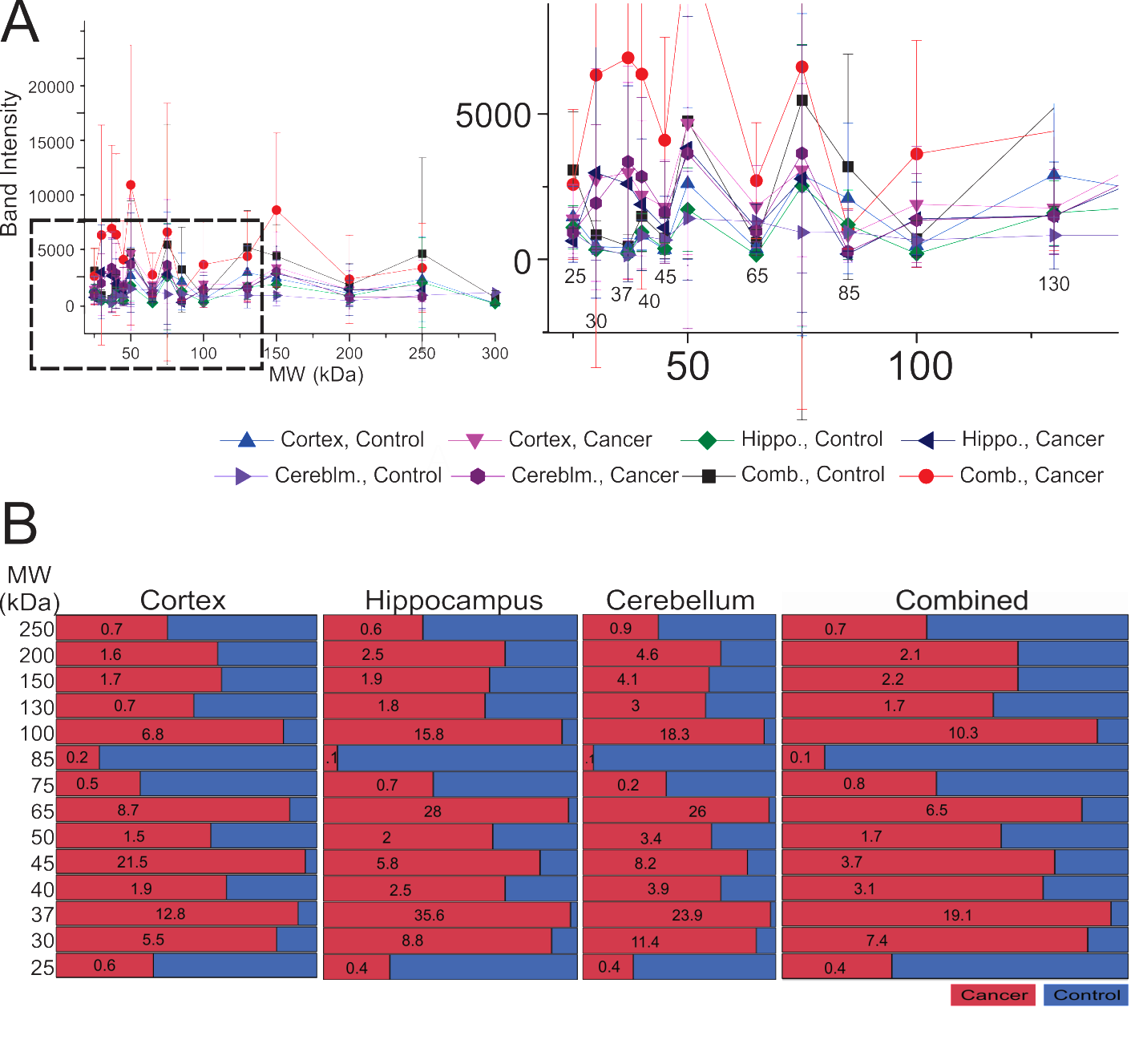
**S1 File. Comparison of Western blot band intensities. A.** The average (mean ± SD) band intensities at 25-300 kDa molecular weights were plotted for cortex, hippocampus, and cerebellum individually and in combination (combined). Right panel is a portion of the graph (left, dotted rectangle) zoomed for a closer look at the crowded points at lower molecular weights. Selected molecular weights were plotted in Fig. 6B. **B**. The sum of the band intensities measured at 25 to 250 kDa was plotted for cortex, hippocampus, cerebellum, and total (combined). Intensity folds compared to control are provided in the cancer bar of the graph. 30, 37, 45, 65, and 100 kDa had highest intensity compared to control group, and thus, were selected for further analysis (see Figs, 6 & 7). Hippo., hippocampus; Cereblm., cerebellum; Comb., combined.
