# Supplementary material for: Detection of brain-directed autoantibodies in the serum of non-small cell lung cancer patients
Source: PLoS One. 2017 Jul 26;12(7):e0181409. doi: 10.1371/journal.pone.0181409 (PMC5528996; doi:10.1371/journal.pone.0181409)
Supplement: S1 Table — NMDAR-NR1: N-methyl-D-aspartate receptor-subunit NR1, GABABR: Gamma amino butyric acid B receptor, AMPAR: α-amino-3-hydroxy-5-methyl-4-isoxazolepropionic acid receptor, GluR: Glutamate receptor, DPPX: Dipeptidyl-peptidase-like protein 6, mGLuR1: metabotropic glutamate receptor 1, DNER: Delta/Notch-like EGF repeat, LGI-1: Leucin-rich glioma inactivated 1 protein, a protein associated with voltage-gated potassium channels (VGKC), CRMP5: Collapsin response-mediator protein 5, PKCγ: Protein kinase C gamma, CDR2: Cerebellar degeneration-related protein 2, AChRs: Nicotinic acetylcholine receptors, GAD2: Glutamic acid decarboxylase 2, SYT1: Synaptotagmin 1, ZIC: Zinc fingers of cerebellum, CV2: Crossveinless 2, Hu: a group of RNA-binding proteins (HuA-HuD), PNMA1/2 (Ma): Pareneoplastic Ma Proteins, AQP-4: Aquaporin-4, MBP: Myelin basic protein, SOX-1: Sex determining region Y-like high mobility group box 1 protein, PTPRN: Protein tyrosine phosphatase receptor type N, ANNA-1: Anti-neuronal nuclear antibody type I, PCA-1: Purkinje cell autoantibodies, Pre.: Predicted molecular weight, Obs.: Observed molecular weight. (DOC) [file pone.0181409.s002.doc]

| **S1 Table. Candidate serum autoantibody targets in brain** | | | | |
| --- | --- | --- | --- | --- |
| **MW**  **(Western blot)** | **Autoantibody Targets** | **Syndrome Associated** | **Remarks**  **(Expression region/cell)** | **Support** |
| **100 kDa** | **NMDAR-NR1** (120 kDa) | Limbic encephalitis | Hippocampus (stratum moleculare), cerebellum | [1] |
|  | **GABA_B_R** (109 kDa) | Limbic encephalitis | Hippocampus (stratum moleculare), cerebellum | [2] |
|  | **AMPAR** (GluR 2/3/4) (100 kDa) | Limbic encephalitis | Hippocampus, cerebellum | [3] |
|  | **DPPX** (107, 115 k Da) |  | Neurons, a component of neuronal A-type K^+^ channels | [4] |
|  | **mGluR1** (Pre. 132 kDa; Obs. 102, 54 kDa) |  | Purkinje cells, cytoplasm | [5] |
|  | **DNER** (Tr) (Pre. 72; Obs. 100 k Da) | Cerebellar degeneration | Purkinje cells (preferentially in dendrite), trans membrane protein | [6] |
|  | **LGI-1** (64 kDa) | Limbic encephalitis | Hippocampus (stratum moleculare), cerebellum | [7, 8] |
| **65 kDa** | **CRMP5** (Obs. 62-70 kDa) | Limbic encephalitis, Encephalomyelitis | Hippocampus, cerebellum (Purkinje cells), cytoplasm | [9] |
|  | **PKCγ** (Pre. 78 kDa; Obs. 34, 40, 66, 78 kDa) |  | Purkinje cells, cytoplasm | [10] |
|  | **CDR2** (Yo) (62, 34 kDa) | Cerebellar degeneration | Purkinje cells, cytoplasm | [11] |
|  | **AChRs** (Pre. 68; Obs. 71 kDa) |  |  | [12] |
|  | **GAD2** (59-65 kDa) | Limbic encephalitis | Cerebellum, hippocampus, cytoplasm | [13] |
|  | **SYT1** (Pre. 48 kDa; Obs. 47, 65 kDa) |  |  | [14] |
|  | **ZIC-2** (Pre. 48 kDa; Obs. 47, 65 kDa) |  | Nucleus | [15] |
|  | **CV2** (66 kDa) | Cerebellar degeneration, Retinopathy, Limbic encephalitis | Cerebellum, hippocampus (stratum moleculare) | [16, 17] |
| **45 kDa** | **Hu** B/C/D (Pre. 40, 42 kDa; Obs. 35-40 kDa) | Cerebellar degeneration, Encephalomyelitis, Limbic encephalitis, neuropathy | Hippocampus, cerebellum, nucleus | [18, 19] |
|  | **PNMA1/2 (Ma)** (40, 41 kDa) | Limbic encephalitis | Hippocampus, cerebellum, neurons, nucleus | [20] |
|  | **α-Enolase** (47 kDa) |  | Nucleus | [21] |
|  | **ZIC-4** (37 kDa) |  | Nucleus | [15] |
|  | **AQP-4** (Pre. 35 kDa; Obs. 48 kDa) |  | Small brain vessels, pia, subpia, Virchow-Robin space on the tissues optic nerve, and cerebellum, integral membrane protein | [22] |
| **37 kDa** | **Myelin** (25 kDa); MBP (Pre. 33 kDa; Obs. 23, 18 kDa) |  | MBP-binds myelin | [11] |
|  | **SOX-1** (Pre. 39 kDa; Obs. 40 kDa) |  |  | [23] |
| **30 kDa** | **PTPRN** (23 kDa) |  |  | [24] |
|  | **Recoverin** (23, 65 kDa) | Retinopathy |  | [21] |
| **NMDAR-NR1**: N-methyl-D-aspartate receptor-subunit NR1, **GABABR**: Gamma amino butyric acid B receptor, **AMPAR**: α-amino-3-hydroxy-5-methyl-4-isoxazolepropionic acid receptor, **GluR**: Glutamate receptor, **DPPX**: Dipeptidyl-peptidase-like protein 6, **mGLuR1**: metabotropic glutamate receptor 1, **DNER**: Delta/Notch-like EGF repeat, **LGI-1**: Leucin-rich glioma inactivated 1 protein, a protein associated with voltage-gated potassium channels (VGKC), **CRMP5**: Collapsin response-mediator protein 5, **PKCγ**: Protein kinase C gamma, **CDR2**: Cerebellar degeneration-related protein 2, **AChRs**: Nicotinic acetylcholine receptors, **GAD2**: Glutamic acid decarboxylase 2, **SYT1**: Synaptotagmin 1, **ZIC**: Zinc fingers of cerebellum, **CV2**: Crossveinless 2, **Hu**: a group of RNA-binding proteins (HuA-HuD), **PNMA1/2 (Ma)**: Pareneoplastic Ma Proteins, **AQP-4**: Aquaporin-4, **MBP**: Myelin basic protein, **SOX-1**: Sex determining region Y-like high mobility group box 1 protein, **PTPRN**: Protein tyrosine phosphatase receptor type N, **ANNA-1**: Anti-neuronal nuclear antibody type I, **PCA-1**: Purkinje cell autoantibodies, **Pre.**: Predicted molecular weight, **Obs.**: Observed molecular weight | | | | |

**References**

1. Dalmau J, Tuzun E, Wu HY, Masjuan J, Rossi JE, Voloschin A, et al. Paraneoplastic anti-N-methyl-D-aspartate receptor encephalitis associated with ovarian teratoma. Ann Neurol. 2007;61(1):25-36. doi: 10.1002/ana.21050. PubMed PMID: 17262855; PubMed Central PMCID: PMCPMC2430743.

2. Lancaster E, Lai M, Peng X, Hughes E, Constantinescu R, Raizer J, et al. Antibodies to the GABA(B) receptor in limbic encephalitis with seizures: case series and characterisation of the antigen. Lancet Neurol. 2010;9(1):67-76. doi: 10.1016/S1474-4422(09)70324-2. PubMed PMID: 19962348; PubMed Central PMCID: PMCPMC2824142.

3. Lai M, Hughes EG, Peng X, Zhou L, Gleichman AJ, Shu H, et al. AMPA receptor antibodies in limbic encephalitis alter synaptic receptor location. Ann Neurol. 2009;65(4):424-34. doi: 10.1002/ana.21589. PubMed PMID: 19338055; PubMed Central PMCID: PMCPMC2677127.

4. Suchecki S, Risley L, Zimmerman E, Molho E, Hanspal E, Dalmau J. A Rare Autoimmune Encephalitis - Due to Dipeptidyl-Peptidase-Like Protein-6 (DPPX) Autoantibody; A Case Report (P5.169). Neurology. 2014;82(10 Supplement).

5. Marignier R, Chenevier F, Rogemond V, Sillevis Smitt P, Renoux C, Cavillon G, et al. Metabotropic glutamate receptor type 1 autoantibody-associated cerebellitis: a primary autoimmune disease? Arch Neurol. 2010;67(5):627-30. doi: 10.1001/archneurol.2010.51. PubMed PMID: 20457964.

6. Greene M, Lai Y, Dalmau J, Lancaster E. Confirmed: The Antigen Of Paraneoplastic Antibodies Previously Named “anti-Tr” Is Delta/Notch-Like Epidermal Growth Factor-Related Receptor (DNER) (S52.005). Neurology. 2014;82(10 Supplement).

7. Ohkawa T, Fukata Y, Yamasaki M, Miyazaki T, Yokoi N, Takashima H, et al. Autoantibodies to epilepsy-related LGI1 in limbic encephalitis neutralize LGI1-ADAM22 interaction and reduce synaptic AMPA receptors. J Neurosci. 2013;33(46):18161-74. doi: 10.1523/JNEUROSCI.3506-13.2013. PubMed PMID: 24227725; PubMed Central PMCID: PMCPMC3828467.

8. Graus F, Dalmau J, Valldeoriola F, Ferrer I, Rene R, Marin C, et al. Immunological characterization of a neuronal antibody (anti-Tr) associated with paraneoplastic cerebellar degeneration and Hodgkin's disease. J Neuroimmunol. 1997;74(1-2):55-61. PubMed PMID: 9119979.

9. Tada S, Furuta M, Fukada K, Hirozawa D, Matsui M, Aoike F, et al. Severe parkinsonism associated with anti-CRMP5 antibody-positive paraneoplastic neurological syndrome and abnormal signal intensity in the bilateral basal ganglia. J Neurol Neurosurg Psychiatry. 2015. doi: 10.1136/jnnp-2015-311569. PubMed PMID: 26374701.

10. Sabater L, Bataller L, Carpentier AF, Aguirre-Cruz ML, Saiz A, Benyahia B, et al. Protein kinase Cgamma autoimmunity in paraneoplastic cerebellar degeneration and non-small-cell lung cancer. J Neurol Neurosurg Psychiatry. 2006;77(12):1359-62. doi: 10.1136/jnnp.2006.097188. PubMed PMID: 16801349; PubMed Central PMCID: PMCPMC2077410.

11. Dalmau J, Rosenfeld MR. Paraneoplastic syndromes of the CNS. Lancet Neurol. 2008;7(4):327-40. doi: S1474-4422(08)70060-7 [pii];10.1016/S1474-4422(08)70060-7 [doi].

12. Vernino S. Neuronal acetylcholine receptor autoimmunity. Ann N Y Acad Sci. 2008;1132:124-8. doi: 10.1196/annals.1405.011. PubMed PMID: 18567861.

13. Saiz A, Blanco Y, Sabater L, Gonzalez F, Bataller L, Casamitjana R, et al. Spectrum of neurological syndromes associated with glutamic acid decarboxylase antibodies: diagnostic clues for this association. Brain. 2008;131(Pt 10):2553-63. doi: 10.1093/brain/awn183. PubMed PMID: 18687732.

14. Takamori M, Takahashi M, Yasukawa Y, Iwasa K, Nemoto Y, Suenaga A, et al. Antibodies to recombinant synaptotagmin and calcium channel subtypes in Lambert-Eaton myasthenic syndrome. J Neurol Sci. 1995;133(1-2):95-101. PubMed PMID: 8583238.

15. Sabater L, Bataller L, Suarez-Calvet M, Saiz A, Dalmau J, Graus F. ZIC antibodies in paraneoplastic cerebellar degeneration and small cell lung cancer. J Neuroimmunol. 2008;201-202:163-5. doi: 10.1016/j.jneuroim.2008.01.018. PubMed PMID: 18639938; PubMed Central PMCID: PMCPMC2582201.

16. Monstad SE, Nostbakken JK, Vedeler CA. CRMP5 antibodies found in a patient with limbic encephalitis and myasthenia gravis. J Neurol Neurosurg Psychiatry. 2009;80(2):241-2. doi: 10.1136/jnnp.2008.149336. PubMed PMID: 19151024.

17. Monstad SE, Drivsholm L, Skeie GO, Aarseth JH, Vedeler CA. CRMP5 antibodies in patients with small-cell lung cancer or thymoma. Cancer Immunol Immunother. 2008;57(2):227-32. doi: 10.1007/s00262-007-0369-1. PubMed PMID: 17657489.

18. Graus F, Keime-Guibert F, Rene R, Benyahia B, Ribalta T, Ascaso C, et al. Anti-Hu-associated paraneoplastic encephalomyelitis: analysis of 200 patients. Brain. 2001;124(Pt 6):1138-48. PubMed PMID: 11353730.

19. Dalmau J, Graus F, Rosenblum MK, Posner JB. Anti-Hu--associated paraneoplastic encephalomyelitis/sensory neuronopathy. A clinical study of 71 patients. Medicine (Baltimore). 1992;71(2):59-72. PubMed PMID: 1312211.

20. Rosenfeld MR, Eichen JG, Wade DF, Posner JB, Dalmau J. Molecular and clinical diversity in paraneoplastic immunity to Ma proteins. Ann Neurol. 2001;50(3):339-48. PubMed PMID: 11558790.

21. Adamus G, Ren G, Weleber RG. Autoantibodies against retinal proteins in paraneoplastic and autoimmune retinopathy. BMC Ophthalmol. 2004;4:5. doi: 10.1186/1471-2415-4-5. PubMed PMID: 15180904; PubMed Central PMCID: PMCPMC446200.

22. Jarius S, Paul F, Franciotta D, Waters P, Zipp F, Hohlfeld R, et al. Mechanisms of disease: aquaporin-4 antibodies in neuromyelitis optica. Nat Clin Pract Neurol. 2008;4(4):202-14. doi: 10.1038/ncpneuro0764. PubMed PMID: 18334978.

23. Tschernatsch M, Gross O, Kneifel N, Kaps M, Blaes F. SOX-1 autoantibodies in patients with paraneoplastic neurological syndromes. Autoimmun Rev. 2009;8(7):549-51. doi: 10.1016/j.autrev.2009.01.015. PubMed PMID: 19393214.

24. Kazarian M, Laird-Offringa IA. Small-cell lung cancer-associated autoantibodies: potential applications to cancer diagnosis, early detection, and therapy. Mol Cancer. 2011;10:33. doi: 10.1186/1476-4598-10-33. PubMed PMID: 21450098; PubMed Central PMCID: PMCPMC3080347.
