# Supplementary material for: Detection of brain-directed autoantibodies in the serum of non-small cell lung cancer patients
Source: PLoS One. 2017 Jul 26;12(7):e0181409. doi: 10.1371/journal.pone.0181409 (PMC5528996; doi:10.1371/journal.pone.0181409)
Supplement: S2 Table — (DOC) [file pone.0181409.s003.doc]

| **S2 Table. Protein sequence comparison between human and rat autoantibody targets listed in S1 Table** | | | | |
| --- | --- | --- | --- | --- |
| **Protein Targets** | **Accession-Human** | **Accession-Rat** | **Length (aa)** | **% Identity** |
| **NMDA-NR1** | NP_001172020.1 | NP_058706.1 | H: 906, R: 938 | 97 |
| **GABA_B_R**ρ | NP_001099050.1 | NP_058987.2 | H: 480, R: 467 | 67 |
| **AMPAR (GluR2)** | NP_001273767.1 | NP_001077280.1 | H: 912, R: 883 | 32 |
| **DPPX** | NP_001277181.1 | NP_074041.1 | H: 758, R: 859 | 98 |
| **mGluR1** | NP_001264996.1 | NP_001107802.1 | H: 908, R: 906 | 98 |
| **DNER (Tr)** | NP_620711.3 | XP_008756285.1 | H: 737, R: 656 | 92 |
| **LGI-1** | NP_005088.1 | NP_665712.1 | H: 557, R: 557 | 97 |
| **CRMP5** | NP_001240653.1 | NP_075412.1 | H: 564, R: 564 | 98 |
| **PKCγ** | NP_001303258.1 | NP_036760.1 | H: 710, R: 697 | 99 |
| **CDR2 (Yo)** | NP_001793.1 | NP_001020853.1 | H: 454, R: 453 | 88 |
| **AChRα** | NP_000737.1 | NP_036964.3 | H: 502, R: 502 | 94 |
| **GAD2** | NP_001127838.1 | NP_036695.1 | H: 585, R: 585 | 96 |
| **SYT1** | NP_001278830.1 | NP_001028852.2 | H: 419, R: 421 | 97 |
| **ZIC-2** | NP_009060.2 | NP_001101862.2 | H: 532, R: 529 | 98 |
| **CV2** | NP_597725.1 | NP_001129271.1 | H: 685, R: 685 | 93 |
| **HuA** | NP_001410.2 | NP_001102318.1 | H: 326, R: 326 | 98 |
| **PNMA1 (Ma)** | NP_006020.4 | NP_570833.1 | H: 353, R: 353 | 94 |
| **Α-Enolase** | NP_001419.1 | XP_006239506.1 | H: 434, R: 434 | 92 |
| **ZIC-4** | NP_001161850.1 | NP_001101646.1 | H: 384, R: 74 | 45 |
| **AQP-4** | NP_001304313.1 | NP_001304678.1 | H: 352, R: 352 | 93 |
| **MBP (MP2)** | NP_002668.1 | NP_001102984.1 | H: 132, R: 132 | 90 |
| **SOX-1** | NP_005977.2 | XP_003752300.1 | H: 391, R: 181 | 99 |
| **PTPRN** | NP_002837.1 | NP_446333.1 | H: 979, R: 983 | 87 |
| **Recoverin** | NP_002894.1 | NP_543177.1 | H: 200, R: 202 | 89 |
