# Supplementary material for: Detection of brain-directed autoantibodies in the serum of non-small cell lung cancer patients
Source: PLoS One. 2017 Jul 26;12(7):e0181409. doi: 10.1371/journal.pone.0181409 (PMC5528996; doi:10.1371/journal.pone.0181409)
Supplement: S3 Table — (DOC) [file pone.0181409.s004.doc]

| **S3 Table. Identification of epitope(s) in human and rat target proteins listed in S1 Table** | | | | |
| --- | --- | --- | --- | --- |
| **Targets** | **% Identity**  **(Human vs Rat)** | **Epitope (Human)** | **Remarks** | **Reference** |
| **NMDA-NR1** | 97 | YSHQSSVWFEMMR |  | [1] |
| **PKCγ** | 99 | ARFFKQPTF |  | [2] |
| **DR2 (Yo)** | 88 | NTELEDSVQQMY | In rat, V>L | [3] |
| **AChRα** | 94 | YKELVKNYNPLERPVAND |  | [4] |
| **GAD2** | 96 | MASPGSGFWSFGSEDGSGDS | In rat, S>P | [5, 6] |
|  |  | TARAWCQVAQKF |  | [7] |
|  |  | KLCALLYGDAEK | In rat, A>S | [7] |
| **HuA** | 98 | GRTNLIVNY |  | [2, 8] |
|  |  | LGYGFVNYV |  | [9] |
| **PNMA1 (Ma)** | 94 | TLQAAMPQV |  | [10] |
| **Α-Enolase** | 92 | KIHAREIFDSRGNPTVE |  | [11] |
|  |  | MRIGAEVYHNL |  | [8] |
| **AQP-4** | 93 | GKCGPLCTRENIMVAFKGVW | In rat, L>P, T>S, N>S | [12] |
|  |  | YTGASMNPARSFGPAVIMGNWENHWI | Identified in rat, but also present in human | [13] |
| **MBP (MP2)** | 90 | ENFDDYMKALGV | In rat, N>H | [14] |
| **PTPRN** | 87 | SEHIWCEDFLVRSFYLKNVQ |  | [15] |
|  |  | LAKEWQALCAYQAEPNTCATAQGEGNIK | In rat, E>K | [15] |
| **Recoverin** | 89 | GTLANKEIL | Identified in mouse, but also present in human and rat | [16] |

**References**

1. Gleichman AJ, Spruce LA, Dalmau J, Seeholzer SH, Lynch DR. Anti-NMDA receptor encephalitis antibody binding is dependent on amino acid identity of a small region within the GluN1 amino terminal domain. J Neurosci. 2012;32(32):11082-94. doi: 10.1523/JNEUROSCI.0064-12.2012. PubMed PMID: 22875940; PubMed Central PMCID: PMCPMC3430387.

2. Caron E, Espona L, Kowalewski DJ, Schuster H, Ternette N, Alpizar A, et al. An open-source computational and data resource to analyze digital maps of immunopeptidomes. Elife. 2015;4. doi: 10.7554/eLife.07661. PubMed PMID: 26154972; PubMed Central PMCID: PMCPMC4507788.

3. Giam K, Ayala-Perez R, Illing PT, Schittenhelm RB, Croft NP, Purcell AW, et al. A comprehensive analysis of peptides presented by HLA-A1. Tissue Antigens. 2015;85(6):492-6. doi: 10.1111/tan.12565. PubMed PMID: 25880248.

4. Shelukhina IV, Kryukova EV, Skok MV, Lykhmus EY, Zhmak MN, Mordvintsev DY, et al. Analysis of specificity of antibodies against synthetic fragments of different neuronal nicotinic acetylcholine receptor subunits. Biochemistry (Mosc). 2006;71(7):749-58. PubMed PMID: 16903829.

5. Ramirez-Montealegre D, Chattopadhyay S, Curran TM, Wasserfall C, Pritchard L, Schatz D, et al. Autoimmunity to glutamic acid decarboxylase in the neurodegenerative disorder Batten disease. Neurology. 2005;64(4):743-5. doi: 10.1212/01.WNL.0000151973.08426.7E. PubMed PMID: 15728308.

6. Yang J, James EA, Sanda S, Greenbaum C, Kwok WW. CD4+ T cells recognize diverse epitopes within GAD65: implications for repertoire development and diabetes monitoring. Immunology. 2013;138(3):269-79. doi: 10.1111/imm.12034. PubMed PMID: 23228173; PubMed Central PMCID: PMCPMC3573280.

7. Rharbaoui F, Mayer A, Granier C, Bouanani M, Thivolet C, Pau B, et al. T cell response pattern to glutamic acid decarboxylase 65 (GAD65) peptides of newly diagnosed type 1 diabetic patients sharing susceptible HLA haplotypes. Clin Exp Immunol. 1999;117(1):30-7. PubMed PMID: 10403912; PubMed Central PMCID: PMCPMC1905465.

8. Schittenhelm RB, Sian TC, Wilmann PG, Dudek NL, Purcell AW. Revisiting the arthritogenic peptide theory: quantitative not qualitative changes in the peptide repertoire of HLA-B27 allotypes. Arthritis Rheumatol. 2015;67(3):702-13. doi: 10.1002/art.38963. PubMed PMID: 25418920.

9. Bourcier KD, Lim DG, Ding YH, Smith KJ, Wucherpfennig K, Hafler DA. Conserved CDR3 regions in T-cell receptor (TCR) CD8(+) T cells that recognize the Tax11-19/HLA-A*0201 complex in a subject infected with human T-cell leukemia virus type 1: relationship of T-cell fine specificity and major histocompatibility complex/peptide/TCR crystal structure. J Virol. 2001;75(20):9836-43. doi: 10.1128/JVI.75.20.9836-9843.2001. PubMed PMID: 11559817; PubMed Central PMCID: PMCPMC114556.

10. Hassan C, Kester MG, de Ru AH, Hombrink P, Drijfhout JW, Nijveen H, et al. The human leukocyte antigen-presented ligandome of B lymphocytes. Mol Cell Proteomics. 2013;12(7):1829-43. doi: 10.1074/mcp.M112.024810. PubMed PMID: 23481700; PubMed Central PMCID: PMCPMC3708169.

11. Hensvold AH, Magnusson PK, Joshua V, Hansson M, Israelsson L, Ferreira R, et al. Environmental and genetic factors in the development of anticitrullinated protein antibodies (ACPAs) and ACPA-positive rheumatoid arthritis: an epidemiological investigation in twins. Ann Rheum Dis. 2015;74(2):375-80. doi: 10.1136/annrheumdis-2013-203947. PubMed PMID: 24276366.

12. Varrin-Doyer M, Spencer CM, Schulze-Topphoff U, Nelson PA, Stroud RM, Cree BA, et al. Aquaporin 4-specific T cells in neuromyelitis optica exhibit a Th17 bias and recognize Clostridium ABC transporter. Ann Neurol. 2012;72(1):53-64. doi: 10.1002/ana.23651. PubMed PMID: 22807325; PubMed Central PMCID: PMCPMC3405197.

13. Zeka B, Hastermann M, Hochmeister S, Kogl N, Kaufmann N, Schanda K, et al. Highly encephalitogenic aquaporin 4-specific T cells and NMO-IgG jointly orchestrate lesion location and tissue damage in the CNS. Acta Neuropathol. 2015;130(6):783-98. doi: 10.1007/s00401-015-1501-5. PubMed PMID: 26530185; PubMed Central PMCID: PMCPMC4654751.

14. Csurhes PA, Sullivan AA, Green K, Pender MP, McCombe PA. T cell reactivity to P0, P2, PMP-22, and myelin basic protein in patients with Guillain-Barre syndrome and chronic inflammatory demyelinating polyradiculoneuropathy. J Neurol Neurosurg Psychiatry. 2005;76(10):1431-9. doi: 10.1136/jnnp.2004.052282. PubMed PMID: 16170091; PubMed Central PMCID: PMCPMC1739377.

15. McLaughlin KA, Gulati K, Richardson CC, Morgan D, Bodansky HJ, Feltbower RG, et al. HLA-DR4-associated T and B cell responses to specific determinants on the IA-2 autoantigen in type 1 diabetes. J Immunol. 2014;193(9):4448-56. doi: 10.4049/jimmunol.1301902. PubMed PMID: 25225671; PubMed Central PMCID: PMCPMC4502080.

16. Wang M, Bai F, Pries M, Buus S, Prause JU, Nissen MH. Identification of MHC class I H-2 Kb/Db-restricted immunogenic peptides derived from retinal proteins. Invest Ophthalmol Vis Sci. 2006;47(9):3939-45. doi: 10.1167/iovs.06-0133. PubMed PMID: 16936108.
